# Global, regional, and country-specific lifetime risks of osteoarthritis, 1990–2021: a systematic analysis for the global burden of disease study 2021

**DOI:** 10.1186/s41256-025-00419-9

**Published:** 2025-07-22

**Authors:** Xinge Zhang, Chuiguo Huang, Zhaolan Hu, Yejun Tan, Pengfei Wang, Lemei Zhu, Jin Kang

**Affiliations:** 1 Department of Rheumatology and Immunology, The Second Xiangya Hospital of Central South University, Changsha, 410011 China; 2 Department of Medicine and Therapeutics, The Chinese University of Hong Kong, Hong Kong Special Administrative Region, People’s Republic of China; 3 Department of Anesthesiology, The Second Xiangya Hospital, Central South University, Changsha, 410011 Hunan China; 4 School of Mathematics, University of Minnesota Twin Cities, Minneapolis, MN USA; 5 Clinical Medical Research Center for Systemic Autoimmune Diseases in Hunan Province, Changsha, 410011 China; 6 School of Public Health, Changsha Medical University, Changsha, 410219 Hunan China

## Abstract

**Background:**

Osteoarthritis (OA) is a leading cause of disability worldwide, yet global estimates of lifetime risk remain limited. This study aims to provide comprehensive global, regional, and national lifetime risk estimates for OA, including knee, hand, and hip subtypes, from 1990 to 2021.

**Methods:**

Using data from the Global Burden of Disease (GBD) Study 2021, we estimated the lifetime risk of developing OA from age 20 onwards, stratified by sex, location, and Socio-demographic Index (SDI). Average annual percentage change (AAPC) was also calculated. Spatial heterogeneity in 2021 lifetime risks and their AAPCs was evaluated using the Factor Detector of the Geographic Detector method, with q-statistics quantifying the explanatory power of demographic, socioeconomic, health system and OA-related risk factors.

**Results:**

In 2021, the global lifetime risk of OA was 14.21% (95% confidence interval: 14.21%, 14.22%), with knee OA at 9.31% (95% CI: 9.31%, 9.32%), hand OA at 3.45% (95% CI: 3.45%, 3.46%), and hip OA at 0.71% (95% CI: 0.71%, 0.71%). Lifetime risk increased with higher socio-demographic Index (SDI), from 11.62% in low-SDI regions to 16.10% in high-SDI regions. The highest risk was observed in the High-income Asia Pacific region (18.10%), led by the Republic of Korea (21.20%). Between 1990 and 2021, global lifetime OA risk increased with an AAPC of 0.30% (95% CI: 0.30%, 0.30%), with the most notable increases in East Asia (AAPC: 0.53%). Spatial heterogeneity analysis revealed that the historical proportions employed in agriculture and with upper secondary education had the strongest associations with 2021 lifetime risks and their trends (q-statistics up to 0.84), followed by life expectancy, SDI, and health-system indicators.

**Conclusions:**

In 2021, 1 in 7 individuals globally were projected to develop OA, with the highest risks in high-SDI regions. The steady rise in OA risk, especially in East Asia, highlights the need for targeted public health strategies that focus on prevention, early diagnosis, and ensuring equitable access to treatment to mitigate the increasing OA burden.

**Supplementary Information:**

The online version contains supplementary material available at 10.1186/s41256-025-00419-9.

## Introduction

Osteoarthritis (OA) is the most common form of arthritis and a leading cause of pain and disability worldwide, particularly among older adults [1, 2]. It primarily affects the knee, hip, and hand joints, leading to joint pain, stiffness, and loss of function [1]. The global prevalence of OA has been steadily increasing due to factors such as aging populations, obesity, and lifestyle changes. In 2021, an estimated 595 million people (7.6% of the global population) were living with OA [2]. The condition imposes substantial economic costs on both individuals and healthcare systems worldwide. The average annual cost per individual with OA ranges from $700 to $15,600 [3]. In the U.S. alone, the combined direct and indirect costs associated with OA and related disorders averaged $486.4 billion annually [4].

Despite the growing recognition of the global burden of OA, existing epidemiological studies have predominantly focused on prevalence and incidence rates. While these metrics provide snapshots of disease burden at specific points or periods, these metrics do not capture the long-term probability of developing OA. Lifetime risk refers to the probability or likelihood that an individual will develop a specific disease or condition at any point during their remaining lifetime, starting from a given age [5]. Estimating the lifetime risk of OA offers a more intuitive measure for both individuals and healthcare providers. It also highlights the cumulative impact of risk factors, such as age, obesity, and joint injuries, over the lifespan of a person. Moreover, lifetime risk estimates are valuable for predicting future healthcare needs, guiding clinical decision-making, and shaping public health policies by identifying populations at the greatest risk [6–8].

Despite these benefits, existing estimates of the lifetime risk of OA have predominantly been conducted in the U.S. [9–11]. Comprehensive lifetime risk estimates and comparative analyses for OA at global, regional, and national levels are still lacking. Additionally, changes in mortality and incidence rates over time could significantly impact these risk estimates [2]. Understanding temporal trends in lifetime risk is crucial for capturing shifts in the burden of OA, yet data on these trends remain scarce.

The Global Burden of Disease (GBD) 2021 study provides comprehensive global, regional, and national estimates of incidence and mortality for a wide range of diseases and injuries from 1990 to 2021 [12]. Leveraging these extensive, standardized data, this study aims to calculate and compare lifetime risk estimates and trends for OA, including knee, hand, and hip, at global, regional, and national levels. 

## Methods

### Data sources and processing

The GBD study, developed by the World Health Organization and the World Bank, provides comprehensive estimates of mortality and disability for various diseases and injuries worldwide [12]. The GBD 2021 update provides full time-series estimates from 1990 to 2021, including incidence, prevalence, mortality, years lived with disability, and disability-adjusted life years for 371 diseases and injuries. These data are disaggregated by sex, age groups, regions, and countries and territories [13]. The methodologies employed in GBD 2021 have undergone thorough peer-review and are described elsewhere [13].

The GBD 2021 gathered OA data from various sources, including hospital records, death certificates, health registries, and cohort studies. These sources encompassed 98 studies conducted in 46 countries and territories [2, 13]. OA cases were defined as symptomatic OA confirmed radiographically with a Kellgren–Lawrence grade of 2–4 and reported pain for at least 1 month within the past year. Subtypes of OA, including hip, knee, and hand OA, were identified using ICD-9 codes starting with 715 and ICD-10 codes M16 (hip), M17 (knee), and M18 (hand).

To generate age-, sex-, and location-specific estimates of OA incidence and mortality, the GBD 2021 used DisMod-MR 2.1 [13], a Bayesian meta-regression tool designed for disease modelling. This process involved aggregating all available OA data and adjusting for variations in data quality and potential biases, such as underreporting or misclassification. The model integrated various data inputs, including incidence, prevalence, remission, and mortality, to produce consistent estimates. Age-specific estimates were derived by applying age-adjusted incidence and mortality rates. Similarly, sex-specific estimates were generated by stratifying data to capture differences in disease burden between men and women. Location-specific estimates were produced using spatial modelling that accounted for regional factors such as healthcare access, economic status, and the prevalence of risk factors. Additionally, temporal trends were also incorporated to ensure robust estimates, which underwent multiple validation checks and expert reviews [13].

For this study, we utilized GBD 2021 data to obtain annual estimates of population size, incidence, and mortality for total OA and its subtypes (knee, hand, and hip OA) from 1990 to 2021. Ethics approval and informed consent were waived because our analyses did not involve direct patient interaction or the use of identifiable personal information.

### Lifetime risk estimation

To estimate the lifetime risk of developing OA, we used a modified Kaplan–Meier method [14], an approach originally developed by the Framingham Study team [15] and widely applied in various epidemiological studies [5, 14, 16, 17]. Unlike the standard Kaplan–Meier approach, which typically uses follow-up duration as the time variable, this method utilizes age. The primary motivation for this adaptation is that most cohort studies provide incomplete lifetime data due to limited follow-up periods. Additionally, this approach accounts for the competing risk of death, a common source of bias in ageing-related disease research. For instance, the lifetime risk of developing Alzheimer’s disease at age 70 was estimated to be 43% without considering the competing risk of death, but dropped to 7% after adjustment [18]. A detailed description of this method is provided in the Supplemental Material. We calculated the lifetime risk for total OA and its subtypes (knee, hand, and hip OA) from age 20 to the oldest age group (95 years or older), at global, regional, and national levels from 1990 to 2021. Countries and territories were grouped into 21 GBD study regions and further classified into five categories based on the socio-demographic index (SDI), which reflects income, education, and fertility rates [13].

### Temporal trend analysis

Temporal trends in lifetime risk were assessed by estimating the average annual percentage change (AAPC) and 95% confidence intervals (CI) over the study period using a joinpoint regression model. Although annual percentage changes and joinpoints were also calculated, we reported only the AAPC as a concise summary of the overall trend. The lifetime risk was calculated using data from the GBD 2021 dataset, which comprises pooled estimates from multiple countries with large sample sizes, resulting in small standard errors due to substantial population numbers and event counts in each location.

### Spatial heterogeneity analysis

To investigate the spatial heterogeneity of factors influencing OA lifetime risk, a comprehensive spatial analysis was conducted encompassing demographic, socioeconomic, health indicators, and OA-related risk factors. Demographic factors included population size, the proportion of adults aged ≥ 65 years, and the proportion of people living in urban areas. Socioeconomic factors comprised the proportion of individuals employed in agriculture, the proportion with upper secondary education or higher, SDI, universal health coverage (UHC) index, health expenditure per capita, and health expenditure as a percentage of gross domestic product (GDP). Health indicators evaluated were life expectancy and healthy life expectancy. OA-related risk factors included the prevalence of overweight/obesity and the rate of physical inactivity. Data for these variables were sourced from the GBD 2021, World Bank, World Health Organization, and International Labour Organization. For each variable, data from the earliest and latest available years were utilized. Detailed sources, definitions, and the index year of measurement for these risk factors are listed in Table S1.

For the spatial analysis, continuous spatial data on all risk factors were classified into several groups using the optimal spatial discretization method [19].Spatial heterogeneity analysis was performed using the Factor Detector component of the Geographic Detector, a statistical method designed to assess the spatial stratified heterogeneity of a variable and identify the explanatory power of various factors contributing to this heterogeneity [20, 21]. Within this framework, the Factor Detector was utilized to estimate the q-statistic to evaluate the individual impact of each factor on the spatial distribution of OA lifetime risk and its temporal trends indicated by AAPCs. The q-statistic is calculated as follows [22]:

$$q=1-\frac{{\sum }_{h=1}^{L}{N}_{h}{\sigma }_{h}^{2}}{N{\sigma }^{2}}$$

where $L$ stands for the number of strata of the factor, ${N}_{h}$ is the number of observations in stratum $h$ ($h$ = 1, 2, …, $L$), ${\sigma }_{h}^{2}$ is the variance within stratum $h$. $N$ is the total number of observations. and ${\sigma }^{2}$ is the total variance across all regions. The q-statistic ranges from 0 to 1, with a higher value indicating a stronger association between the factor and the spatial distribution of the outcome of interest.

Statistical significance was set at a *p*-value of < 0.05. The joinpoint regression model was performed using the Joinpoint Regression Program (Version 5.3.0; Statistical Research and Applications Branch, National Cancer Institute), and other analyses were conducted using R software, version 4.3.2 (R Foundation for Statistical Computing, Vienna, Austria).

## Results

### Global, regional, and national lifetime risk estimates of osteoarthritis in 2021

In 2021, the global lifetime risk of overall OA was estimated at 14.21% (95% CI: 14.21%, 14.22%). Knee OA was the most affected joint, with a lifetime risk of 9.31% (9.31%, 9.32%). Hand OA followed at 3.45% (3.45%, 3.46%), and hip OA had the lowest lifetime risk at 0.71% (0.71%, 0.71%). The lifetime risk of OA increased with higher SDI levels, ranging from 11.62% (95% CI: 11.59%, 11.65%) in low SDI regions to 16.10% (16.09%, 16.12%) in high SDI regions (Table 1).

**Table 1:** Global and regional expected lifetime risks (%) of osteoarthritis and its subtypes for both sexes combined in 2021

	OA	OA knee	OA hand	OA hip
Global	14.21 (14.21, 14.22)	9.31 (9.31, 9.32)	3.45 (3.45, 3.46)	0.71 (0.71, 0.71)
Low SDI	11.62 (11.59, 11.65)	7.80 (7.78, 7.82)	2.44 (2.42, 2.46)	0.61 (0.60, 0.62)
Low-middle SDI	12.64 (12.62, 12.65)	8.35 (8.34, 8.37)	2.95 (2.94, 2.96)	0.55 (0.55, 0.56)
Middle SDI	14.25 (14.24, 14.27)	9.40 (9.39, 9.41)	3.47 (3.47, 3.48)	0.64 (0.64, 0.65)
High-middle SDI	14.70 (14.69, 14.72)	9.45 (9.44, 9.46)	3.62 (3.61, 3.63)	0.91 (0.90, 0.92)
High SDI	16.10 (16.09, 16.12)	10.40 (10.39, 10.41)	4.14 (4.13, 4.14)	0.94 (0.93, 0.94)
Central Europe, Eastern Europe, and Central Asia	14.79 (14.76, 14.81)	7.75 (7.74, 7.77)	4.82 (4.81, 4.83)	1.65 (1.64, 1.67)
Central Asia	13.41 (13.33, 13.48)	6.68 (6.64, 6.73)	4.60 (4.56, 4.64)	1.47 (1.43, 1.52)
Central Europe	14.45 (14.41, 14.49)	7.81 (7.79, 7.84)	4.27 (4.25, 4.29)	1.73 (1.71, 1.76)
Eastern Europe	15.28 (15.25, 15.31)	8.01 (7.99, 8.03)	5.14 (5.12, 5.16)	1.63 (1.62, 1.65)
High-income	16.14 (16.13, 16.16)	10.53 (10.52, 10.54)	4.04 (4.03, 4.04)	0.94 (0.94, 0.95)
Australasia	16.25 (16.18, 16.33)	10.81 (10.75, 10.87)	3.77 (3.72, 3.82)	0.96 (0.95, 0.98)
High-income Asia Pacific	18.10 (18.08, 18.13)	12.35 (12.33, 12.37)	4.64 (4.62, 4.65)	0.62 (0.62, 0.63)
High-income North America	16.31 (16.29, 16.33)	10.06 (10.04, 10.08)	4.60 (4.59, 4.62)	1.09 (1.08, 1.09)
Southern Latin America	15.32 (15.26, 15.37)	10.21 (10.17, 10.26)	3.57 (3.54, 3.61)	0.85 (0.84, 0.86)
Western Europe	15.10 (15.08, 15.12)	10.01 (10.00, 10.02)	3.37 (3.36, 3.38)	0.98 (0.98, 0.99)
Latin America and Caribbean	15.28 (15.26, 15.31)	9.99 (9.98, 10.01)	3.84 (3.83, 3.85)	0.78 (0.77, 0.79)
Andean Latin America	14.70 (14.64, 14.76)	9.94 (9.89, 9.99)	3.34 (3.30, 3.37)	0.73 (0.71, 0.76)
Caribbean	14.44 (14.37, 14.50)	9.74 (9.69, 9.79)	3.23 (3.19, 3.26)	0.76 (0.73, 0.78)
Central Latin America	15.17 (15.14, 15.21)	9.98 (9.95, 10.00)	3.80 (3.78, 3.82)	0.72 (0.71, 0.73)
Tropical Latin America	15.72 (15.68, 15.76)	10.08 (10.05, 10.11)	4.14 (4.11, 4.16)	0.86 (0.85, 0.87)
North Africa and Middle East	12.94 (12.91, 12.97)	8.58 (8.56, 8.60)	2.79 (2.78, 2.81)	0.71 (0.70, 0.72)
South Asia	13.00 (12.98, 13.02)	8.45 (8.44, 8.47)	3.31 (3.30, 3.32)	0.48 (0.47, 0.48)
Southeast Asia, East Asia, and Oceania	14.18 (14.16, 14.19)	9.68 (9.67, 9.69)	3.15 (3.14, 3.16)	0.57 (0.57, 0.58)
East Asia	14.95 (14.93, 14.96)	10.48 (10.47, 10.49)	3.14 (3.13, 3.15)	0.57 (0.56, 0.58)
Oceania	12.04 (11.80, 12.27)	8.22 (8.04, 8.40)	2.48 (2.34, 2.62)	0.56 (0.47, 0.65)
Southeast Asia	11.89 (11.87, 11.92)	7.35 (7.33, 7.37)	3.14 (3.13, 3.16)	0.57 (0.57, 0.58)
Sub-Saharan Africa	12.27 (12.24, 12.29)	7.90 (7.89, 7.92)	2.87 (2.85, 2.89)	0.78 (0.77, 0.79)
Central Sub-Saharan Africa	11.57 (11.48, 11.66)	7.42 (7.36, 7.48)	2.74 (2.69, 2.80)	0.71 (0.66, 0.75)
Eastern Sub-Saharan Africa	11.77 (11.72, 11.82)	7.55 (7.52, 7.58)	2.74 (2.71, 2.77)	0.77 (0.75, 0.80)
Southern Sub-Saharan Africa	13.42 (13.34, 13.49)	8.15 (8.10, 8.20)	3.73 (3.68, 3.77)	0.93 (0.89, 0.96)
Western Sub-Saharan Africa	12.53 (12.48, 12.58)	8.27 (8.24, 8.30)	2.75 (2.72, 2.78)	0.76 (0.74, 0.78)

**Fig. 1:**
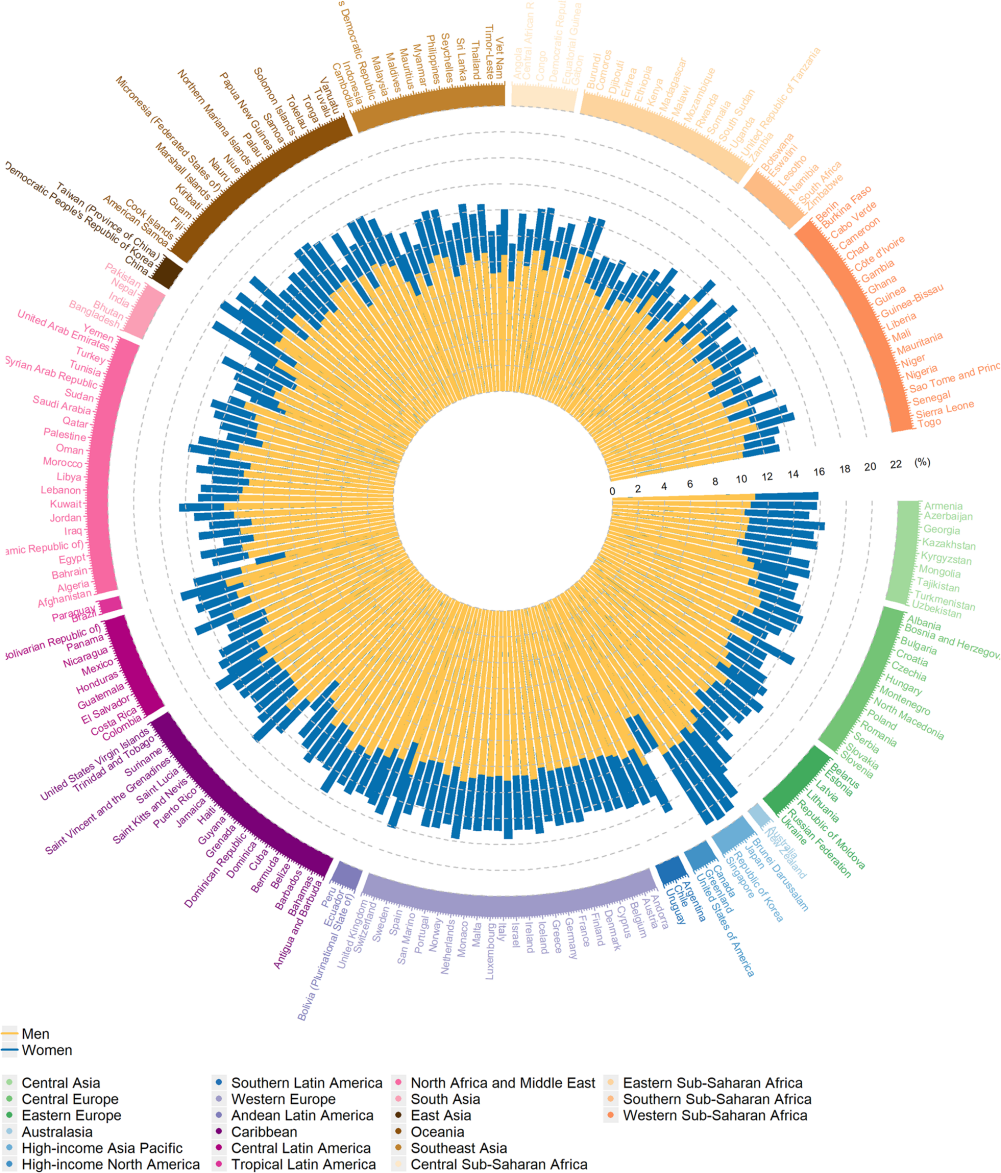
Lifetime risk estimates of osteoarthritis in 2021

The High-income Asia Pacific region exhibited the highest overall lifetime risk of OA at 18.10%, largely driven a lifetime risk of 12.35% for knee OA. For OA subtypes, the highest lifetime risks were observed in Eastern Europe for hand OA (5.14%) and in Central Europe for hip OA (1.73%). In contrast, Sub-Saharan Africa, South Asia, Southeast Asia, East Asia, and Oceania showed the lowest lifetime risks for overall and its subtypes. At the national level, countries within the High-income Asia Pacific region exhibited the highest lifetime risks for total OA, with the Republic of Korea at 21.20% and Japan at 21.09%. For knee OA, the Republic of Korea and Singapore had the highest risks at 15.26% and 14.83%, respectively. Kazakhstan in Central Asia showed the highest lifetime risk for hand OA at 7.33%, followed by Mongolia at 7.31%. For hip OA, Slovenia in Central Europe had the highest risk at 2.03%. (Table 1, Fig. 1, Table S2, and Fig. S1).

Across all OA subtypes, women consistently exhibited higher lifetime risks than men, particularly for knee and hand OA. The largest disparities were found in High-income regions, while the gaps were smaller in South Asia, East Asia, and Sub-Saharan Africa. However, for hip OA, men generally had a higher lifetime risk than women, especially in Eastern Europe and Central Europe (Fig. 1, Fig. S1, Tables S3–4, Table S5–6).

### Temporal trends in lifetime risk estimates

**Fig. 2:**
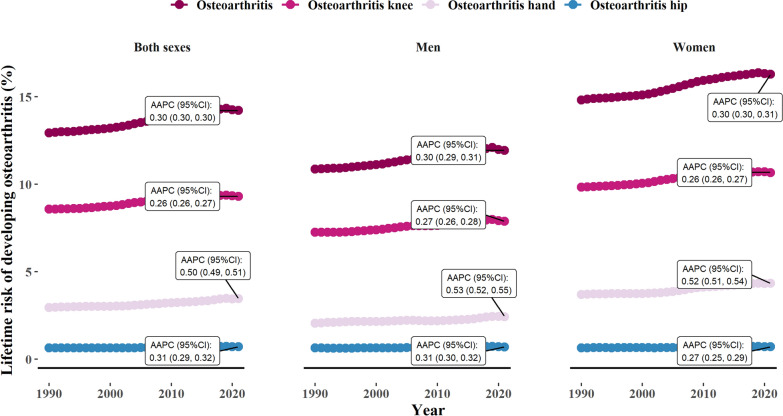
Temporal change in global lifetime risk estimates of osteoarthritis from 1990 to 2021, stratified by sex

From 1990 to 2021, there was a steady global increase in the lifetime risk of different OA types. Total OA lifetime risk rose by an AAPC of 0.30% (95% CI: 0.30%, 0.30%), with knee OA increasing by 0.26% (95% CI: 0.26%, 0.27%), hand OA by 0.50% (95% CI: 0.49%, 0.51%), and hip OA by 0.31% (95% CI: 0.29%, 0.32%). The increase in lifetime risk was generally similar between men and women for both total OA and its subtypes, as indicated by comparable AAPCs (Fig. 2).

**Fig. 3:**
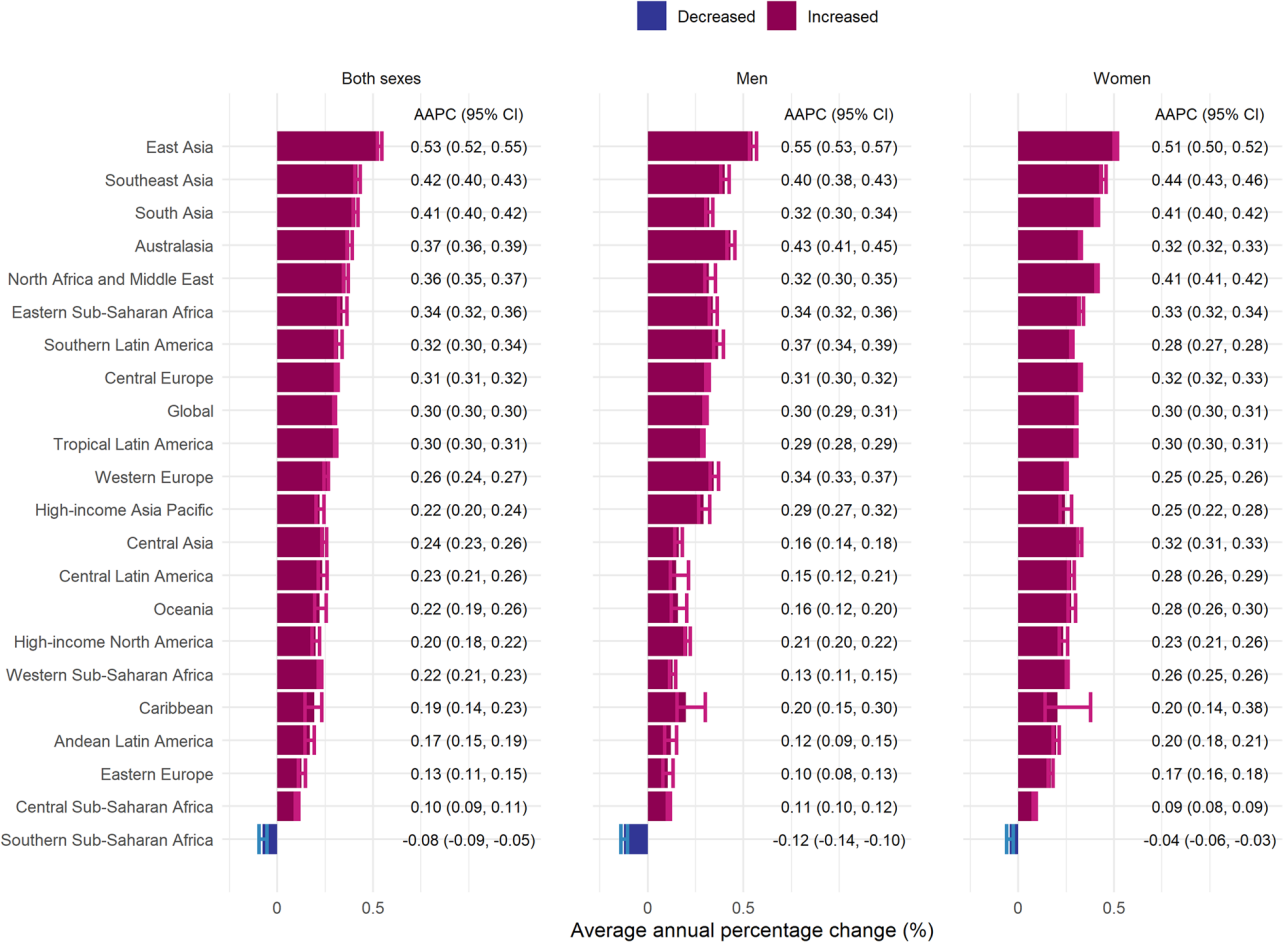
Average annual percentage change in lifetime risk estimates of overall osteoarthritis by region from 1990 to 2021, stratified by sex

Regional trends in the lifetime risk of OA varied. Notable increases in total OA risk were observed in East Asia, Southeast Asia, and South Asia, with AAPCs at 0.53%, 0.42%, and 0.41%, respectively. In contrast, Southern Sub-Saharan Africa experienced a slight decline. Knee OA risk showed prominent increases in Australasia, Southern Lartin America, and East Asia, while Southern Sub-Saharan Africa was the only region with a noticeable decline. Hand OA exhibited the largest rise in East Asia (AAPC: 1.37%) and Eastern Sub-Saharan Africa (AAPC: 0.85%). Hip OA saw the highest increases in East Asia (AAPC: 1.22%) and Australasia (AAPC: 0.93%) (Fig. 3 and Fig. S2–4).

### Spatial heterogeneity analysis

In 2021, the largest variance in lifetime risks of total OA and its subtypes was explained by the proportions of people employed in agriculture and the proportions with at least upper secondary education, both historically and currently (q-statistics ranging from 0.387 to 0.842). Broader indicators, including life expectancy, healthy life expectancy, SDI, health expenditure per capita, and UHC index, also played key roles in explaining spatial patterns of OA lifetime risk. Notably, historical values of these factors generally exhibited higher q-statistics than their contemporary counterparts, underscoring the cumulative effects of long-term exposures on OA lifetime risk.

**Fig. 4:**
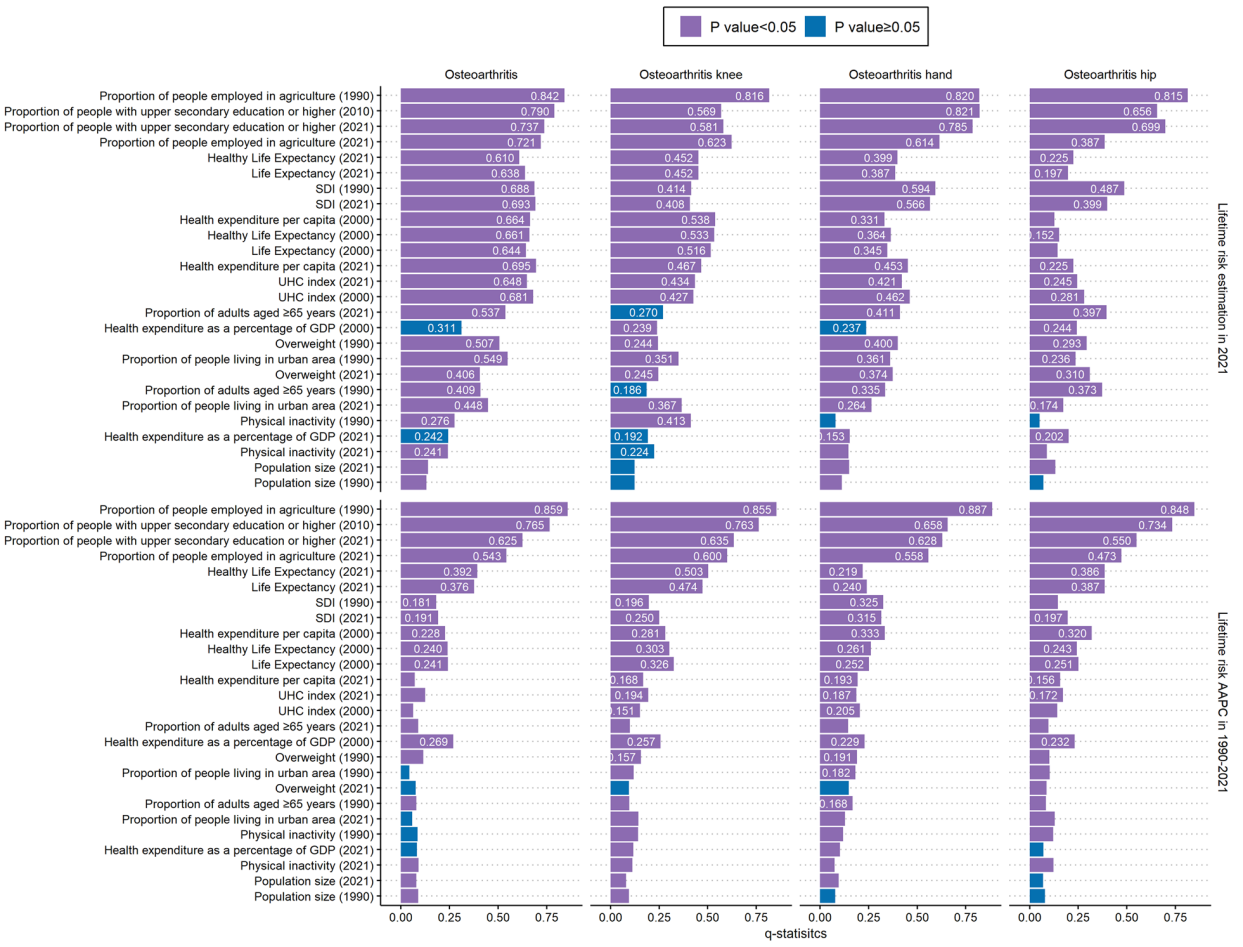
Spatial heterogeneity analysis and spatial drivers of OA lifetime risk. AAPC: average annual percentage change; GBD: Global burden of disease; GDP: gross domestic product; OA: osteoarthritis; SDI: socio-demographic index; UHC: universal health coverage

Similarly, from 1990 to 2021, the proportions of people employed in agriculture and those with at least upper secondary education, especially historical values, were consistently the most influential factors for AAPCs in the lifetime risk of total OA and its subtypes. Life expectancy and healthy life expectancy in 2021 also showed moderate q-statistics (Fig. 4).

## Discussion

This study offers the first comprehensive global, regional, and national estimates of OA lifetime risk for 2021, providing new insights into OA burden beyond the existing literature which has primarily focused on prevalence, incidence, mortality, and disability-adjusted life years using GBD data [2, 23]. Globally, approximately 1 in 7 individuals is expected to develop OA during their lifetime. Knee OA, the primary weight-bearing joint, had the highest lifetime risk at 9.31%, followed by hand OA at 3.45% and hip OA at 0.71%. Regions with higher socioeconomic development, particularly the High-income Asia Pacific, exhibited the highest lifetime risks, while Sub-Saharan Africa, South Asia, and Southeast Asia showed the lowest risks. Our analysis also reveals a steady increase in the global lifetime risk of all OA subtypes from 1990 to 2021, with hand OA displaying the largest growth. Additionally, women consistently showed higher lifetime risks for knee and hand OA. Historical and current proportions of agriculture employment and education levels explained the largest spatial heterogeneity. The potential explanations behind these findings are explored in detail below.

### Comparison with existing evidence

Existing evidence on OA lifetime risk has largely focused on U.S. populations. The Johnston County study of rural North Carolinians aged ≥ 45 years reported a 45% lifetime risk of symptomatic knee OA, with even higher risks among those with a history of knee injury (57%) and obesity (up to 66%) [24]. Another study among younger U.S. adults aged 25–44 years, recruited online, reported a 25% lifetime risk of knee OA [11]. These higher estimates compared to our findings are likely attributable to the inclusion of self-reported cases rather than solely diagnosed OA. Additionally, these populations may not be representative of the broader U.S. population. A nationwide cohort study using 2007–2008 National Health Interview Survey data reported a lower lifetime risk of diagnosed symptomatic knee OA at 13.83%, ranging from 9.60% in nonobese men to 23.87% in obese women [9]. These nationwide estimates are closer to those in our study, suggesting that our lower estimates may more accurately reflect the general population.

Lifetime risk estimates complement traditional OA incidence and prevalence studies [2] by translating annual rates into cumulative lifespan probability, thereby motivating the need for proactive prevention strategies. For instance, OA is commonly perceived as a disease primarily affecting older individuals. Annual knee OA incidence in the U.S. peaks at 0.37% to 1.02% in adults aged 55 to 64 years, and OA prevalence consistently increases with age [9]. However, our analysis reveals that the lifetime risk of developing knee OA for the U.S. population from birth is over 10%. This highlights a different perspective and the need for early education and interventions targeting younger populations. Unlike prevalence or incidence, lifetime risk uniquely illuminates long-term demographic shifts tied to aging and life expectancy. For instance, prior GBD data indicated a decrease in OA burden in Eastern Europe and a stable trend in High-income North America [23]. However, lifetime risk estimates in these regions have increased due to aging demographics and extended lifespans. This divergence highlights that even with stable or declining annual burden metrics, aging societies face a growing proportion of individuals living long enough to accumulate irreversible joint damage.

### Regional variations of OA lifetime risk

The direct drivers of OA lifetime risk are OA incidence and life expectancy. Individuals with higher expected OA incidence and the potential to live longer have a higher lifetime risk of OA. The regional variations in lifetime risk of OA observed in our study underscore the multifactorial nature of OA development and life expectancy, driven by the interplay of genetic, environmental, and lifestyle factors. The High-income Asia Pacific region, with the highest overall lifetime risk of OA at 18%, was largely driven by knee OA at 12%. This elevated risk can be attributed to a rapidly aging population [9], rising obesity rates [25], and lifestyle shifts [26] associated with urbanization, particularly in countries such as the Republic of Korea, Japan, and Singapore, which reported the highest lifetime risks for total OA at 21%. Knee OA was especially prevalent in these regions, with the Republic of Korea, Singapore, and Brunei Darussalam reporting the highest lifetime risks, reaching 15%. In contrast, Central Asia recorded the highest lifetime risk for hand OA at 5%, with Kazakhstan and Mongolia leading. This elevated risk is linked to the high prevalence of manual labour-intensive occupations in the region, aligning with our spatial analysis identifying agriculture as a key driver. High rates of such occupations, including livestock farming, mining, construction, and traditional crafts, involve repetitive hand movements and physical strain that contribute to joint degeneration over time [27–30]. Central Europe recorded the highest lifetime risk for hip OA at 1.7%, with Slovenia reporting the highest national risk at 2%. This can largely be attributed to genetic predispositions, such as the higher prevalence of developmental dysplasia of the hip [31], a condition frequently diagnosed in the region. Additionally, the high prevalence of asymptomatic femoroacetabular impingement (affecting up to 75% of populations of European descent), an abnormal bone growth that causes hip friction, contributes to this risk [32]. Conversely, regions such as Sub-Saharan Africa, South Asia, and Southeast Asia reported the lowest lifetime risks for both total OA and specific types. It is important to recognize that these lower estimates may be influenced by underdiagnosis and limited access to healthcare [33], potentially underrepresenting the true burden of OA in these regions.

### Temporal trends

Despite improvements in several socioeconomic indicators over the past decades, including a decreasing proportion of agricultural employment, increasing health expenditure per capita, expansion of UHC, and a growing proportion of the population living in urban areas, the lifetime risk of OA continues to steadily increase. A primary driver of this trend is the global aging population. As life expectancy rises, a larger proportion of individuals live into older age, a period when the risk of developing OA naturally increases due to cumulative joint wear and tear. Concurrently, rising overweight and obesity rates worldwide exacerbate this trend by placing additional mechanical stress on weight-bearing joints such as the knees and hips, accelerating cartilage degeneration and increasing the likelihood of OA onset. Furthermore, increasing prevalence of physical inactivity leads to weakening muscle support around joints, contributing to joint instability and degeneration. Additionally, advances in medical diagnostics and greater healthcare accessibility over the study period have likely improved the detection and reporting of OA cases, contributing to the observed increase in lifetime risk estimates. These temporal trends in the rising lifetime risk of OA underscore the urgent need for targeted, multifaceted interventions at both population and healthcare levels.

### Implications

This study highlights several implications. First, estimating OA lifetime risk using GBD data complements traditional incidence and prevalence metrics by incorporating aging populations and increasing lifespans. This approach frames OA as a lifelong health threat, stressing the need for early prevention (e.g., obesity control, joint injury avoidance) and sustained education. Its forward-looking perspective can help policymakers prioritize youth education, preventive strategies, and healthcare infrastructure investment. Additionally, presenting OA risk as a lifetime metric enhances public health messaging, fostering proactive adoption of preventive habits (e.g., exercise, ergonomics) before symptom onset. Second, the proportion of agricultural employment and education levels are major determinants of the spatial heterogeneity in lifetime OA risk. Implementing targeted occupational health policies in regions with a high prevalence of manual labour is essential. Educating workers and employers on ergonomics, providing protective equipment, and promoting safer work environments can mitigate repetitive joint strain and reduce OA incidence. Third, our spatial heterogeneity analysis reveals that UHC accounts for over 60% of the regional variations in lifetime risk of total OA. This underscores the importance of equitable access to healthcare services in managing OA risk and reducing health inequalities. Regions with comprehensive UHC are better equipped to provide early diagnosis, effective management, and preventive care, whereby lowering OA incidence and improving long-term outcomes. Future research and policies should focus on enhancing global access to healthcare and rehabilitation services. Fourth, successful large-scale interventions, such as the state-level arthritis programs in the U.S., have demonstrated the effectiveness of community-based initiatives that promote physical activity, joint-friendly exercises, and self-management [34]. Programs such as the Chronic Disease Self-Management Program [35] and Walk With Ease [36] have proven effective in reducing OA symptoms, improving mobility, and enhancing quality of life. Future research should evaluate the cost-effectiveness of these large-scale interventions. Finally, increased investment in research is necessary to deepen our understanding of OA pathophysiology and regional differences.

### Limitations

Several general limitations of the GBD Study apply to our analysis [13, 37]. First, the estimation of lifetime risk relies on location-specific OA incidence and mortality rates. As the GBD synthesizes data from multiple countries and regions with varying demographic and health system, data gaps or scarcity in certain areas, particularly Sub-Saharan Africa, are inevitable [13]. Although GBD applies rigorous statistical models to harmonize heterogeneous data, underrepresentation or inaccuracies for specific populations or subpopulations may still exist. Second, although ICD-9 and ICD-10 codes have been validated for total OA, potential misclassification or underreporting of OA cannot be fully ruled out, especially in low-resource settings. A model to assign specific causes to cases of OA was developed to address this [13], but this approach introduces additional uncertainty due to assumptions and extrapolations. Third, while we accounted for sex-specific differences in lifetime risk, the study did not explore other demographic factors such as socioeconomic status, ethnicity, or comorbidities, which could provide a more nuanced understanding of OA risk across subpopulations. Fourth, the narrow CIs reported in this study are due to the reliance of lifetime risk calculations on large population sizes and event counts for each location. It is important to interpret these narrow CIs with caution, as they do not eliminate the possibility of unmeasured confounders or variability that could influence the estimates, especially across diverse populations. Finally, these projections are based on current trends and do not account for potential future changes in risk factors, healthcare access, or intervention implementation. Thus, the estimates should be interpreted as reflections of historical and current patterns rather than definitive future predictions.

## Conclusions

This study highlights significant global, regional, and national variations in the lifetime risk of OA. In 2021, the global lifetime risk of overall OA was estimated at 14.21%. The knee was the most affected joint, followed by the hand and hip. Regions with higher socioeconomic development, particularly the High-income Asia Pacific, exhibited the highest lifetime risks. Temporal trends from 1990 to 2021 revealed a steady increase in lifetime OA risk, especially in East Asia. These findings underscore the necessity for region-specific public health strategies focusing on prevention, early diagnosis, and equitable access to care.

## Supplementary Information

Additional file 1.

## Data Availability

The datasets used and/or analysed during the current study are available from the corresponding author on reasonable request.
