# Supplementary material for: Global, regional, and country-specific lifetime risks of osteoarthritis, 1990–2021: a systematic analysis for the global burden of disease study 2021
Source: Glob Health Res Policy. 2025 Jul 22;10:29. doi: 10.1186/s41256-025-00419-9 (PMC12281868; doi:10.1186/s41256-025-00419-9)
Supplement: Supplementary file 1 — Additional file 1. [file 41256_2025_419_MOESM1_ESM.docx]

Supplemental method: The modified Kaplan-Meier method

A modified Kaplan–Meier method developed by the Framingham study was also applied to estimate the remaining lifetime risk ^1^. Conventionally, Kaplan-Meier survival analysis takes the follow-up time as the time variable. In the modified method, age at entry into the study is set as the left-truncation variable and survival age (free of chronic liver disease and alive) is used as the time variable.

Individuals who achieved a certain age $j$ free of chronic liver disease at some point during follow-up constituted the population at risk for age $j$ (risk set, $R_{j}$). If an individual progressed to chronic liver disease, died or was censored at age $j$, he or she was removed from the risk set for age $j$ +1 and older. If an individual entered the study at age $j+1$, he or she would be counted into the risk set for age $j+1$. For the lifetime risk at 20 years, hazards ($h_{j}$), age-specific incidences ($f_{j}$), cumulative incidences ($F_{j}$), and survival probabilities ($S_{j}$) were calculated according to the standard Kaplan–Meier method for each age $j$ (assuming $F_{19}$ = 0 and $S_{19}$ = 1):

$h_{j}=e_{j}/R_{j}$ ($e_{j}$ is the sum of incident diabetes at age $j$);

$f_{j}=h_{j}\times S_{j-1}$;

$F_{j}=\sum_{j=20}^{\mathrm{maximum} age} f_{j}$;

$S_{j}=1-F_{j}$.

However, $F_{j}$ was the cumulative incidence that applied to individuals who survive through age $j-1$, which might be biased by the competing risk of death and cause overestimation of the lifetime risk. Therefore, a separate survival function ($U_{j}$) with death was included as an event alongside chronic liver disease to adjust for the competing risk of death. The adjusted lifetime risk was calculated as follows (assuming $F_{19}$ = 0 and $U_{19}$ = 0):

$h_{j}=e_{j}/R_{j}$ ($e_{j}$ is the sum of incident diabetes at age $j$);

$U_{j}=1-\sum_{j=20}^{\mathrm{maximum} age} \left( \frac{c_{j}}{R_{j}} \right)\times U_{j-1}$ ($c_{j}$ is the sum of incident diabetes or death at age j);

$f_{j}=h_{j}\times U_{j-1}$;

$F_{j}=\sum_{j=20}^{\mathrm{maximum} age} f_{j}$;

$S_{j}=1-F_{j}$.

1. Beiser A, D'Agostino RB, Sr., Seshadri S, Sullivan LM, Wolf PA. Computing estimates of incidence, including lifetime risk: Alzheimer's disease in the Framingham Study. The Practical Incidence Estimators (PIE) macro. *Stat Med* 2000; **19**(11-12): 1495-522.

Fig. S1. Lifetime risk estimates of osteoarthritis in knee, hand, and hip in 2021.


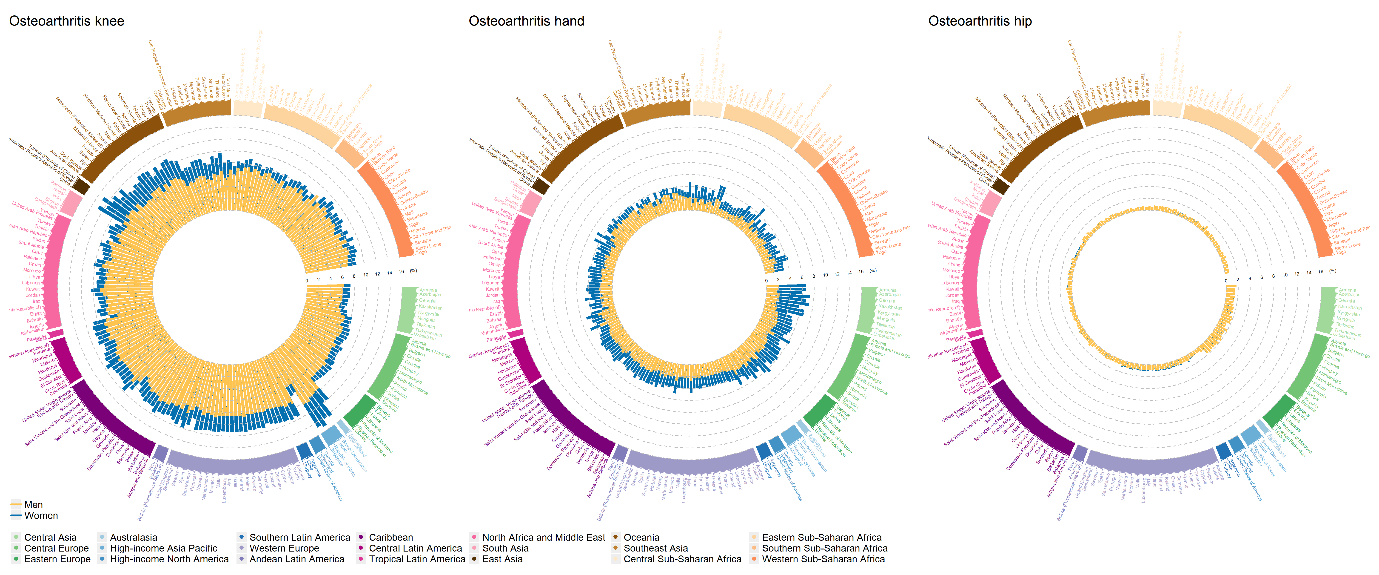


Fig. S2. Average annual percentage change in lifetime risk estimates of specific osteoarthritis by region from 1990 to 2021, in both sexes.


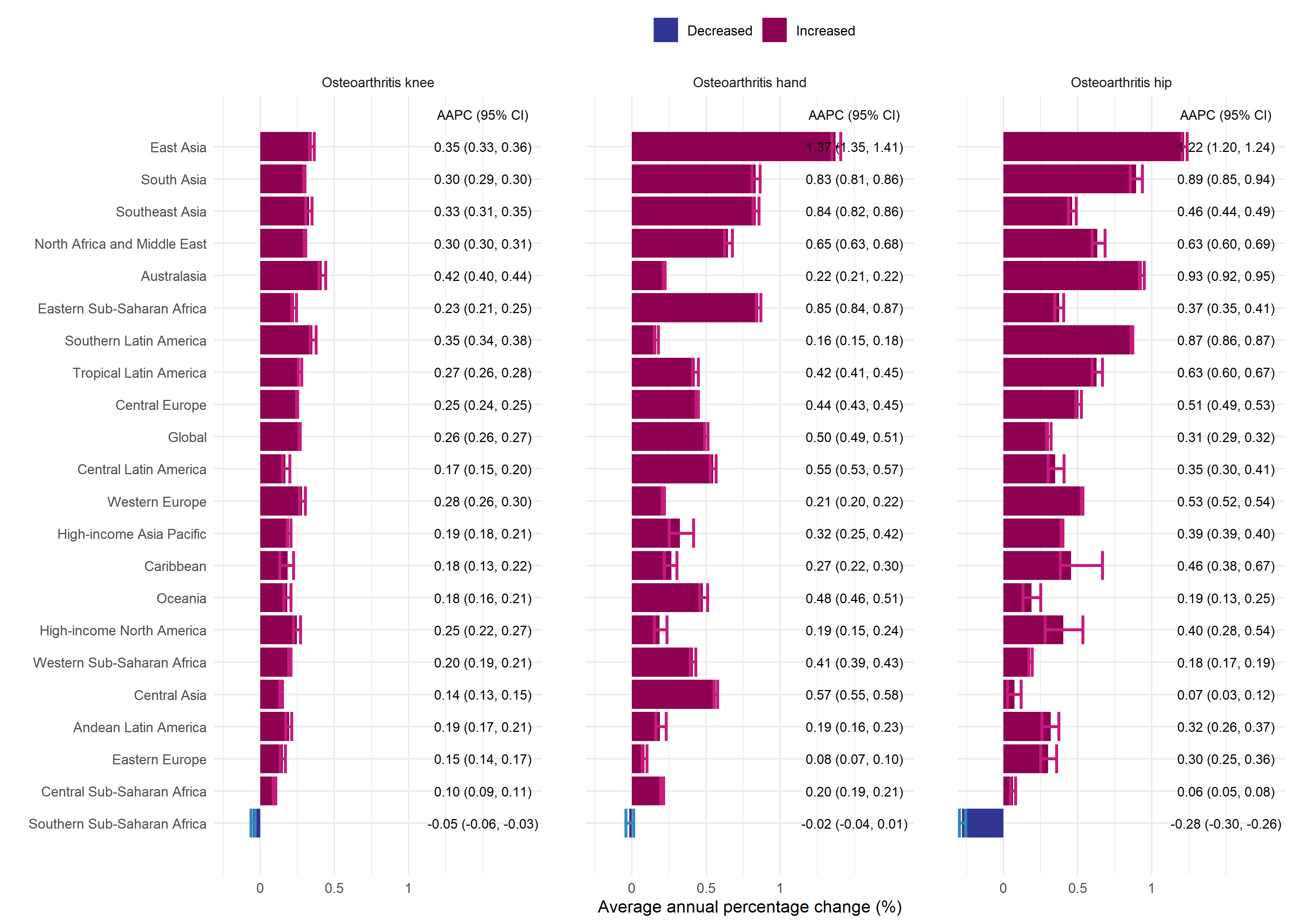


Fig. S3. Average annual percentage change in lifetime risk estimates of specific osteoarthritis by region from 1990 to 2021, in men.


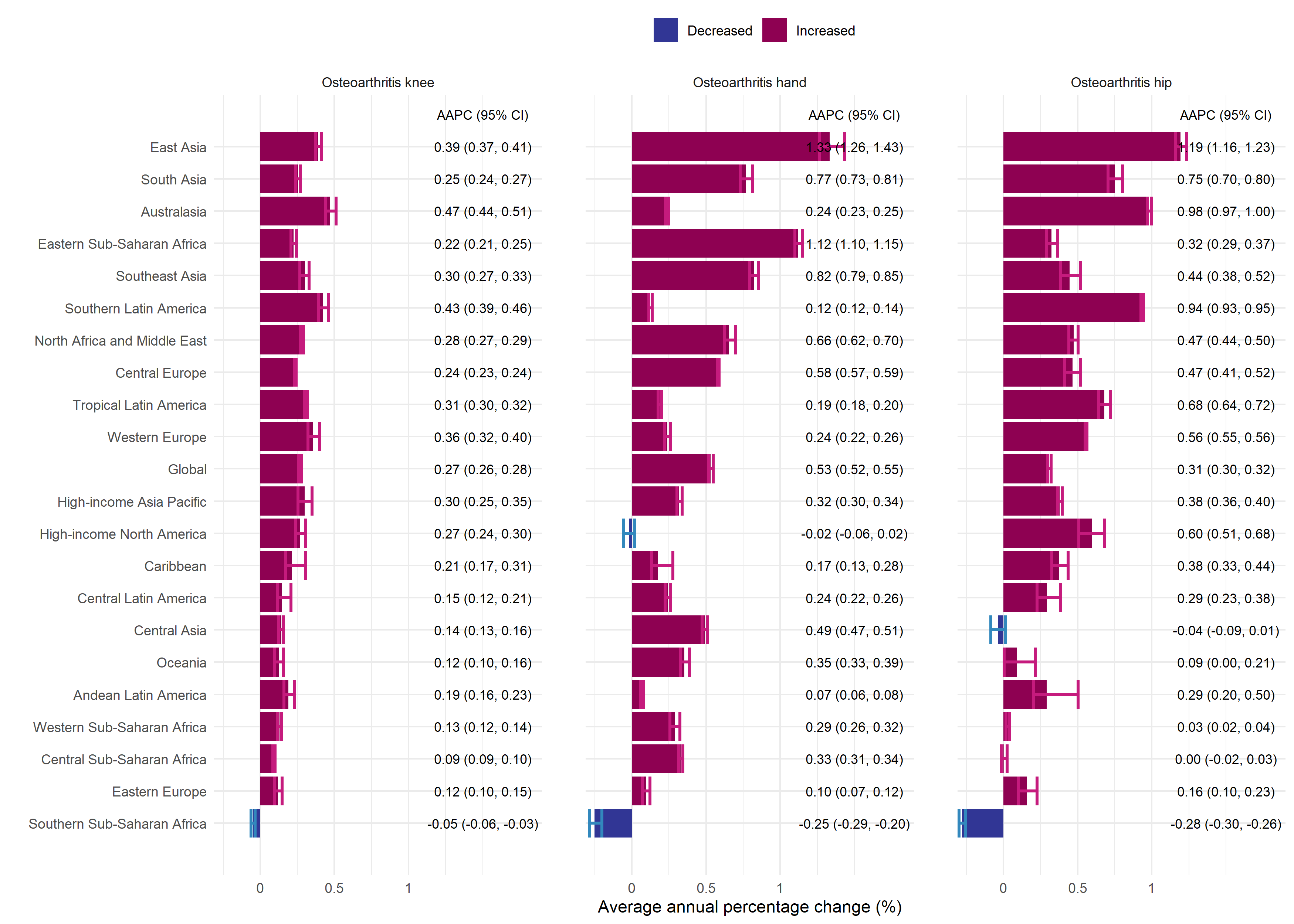


Fig. S4. Average annual percentage change in lifetime risk estimates of specific osteoarthritis by region from 1990 to 2021, in women.


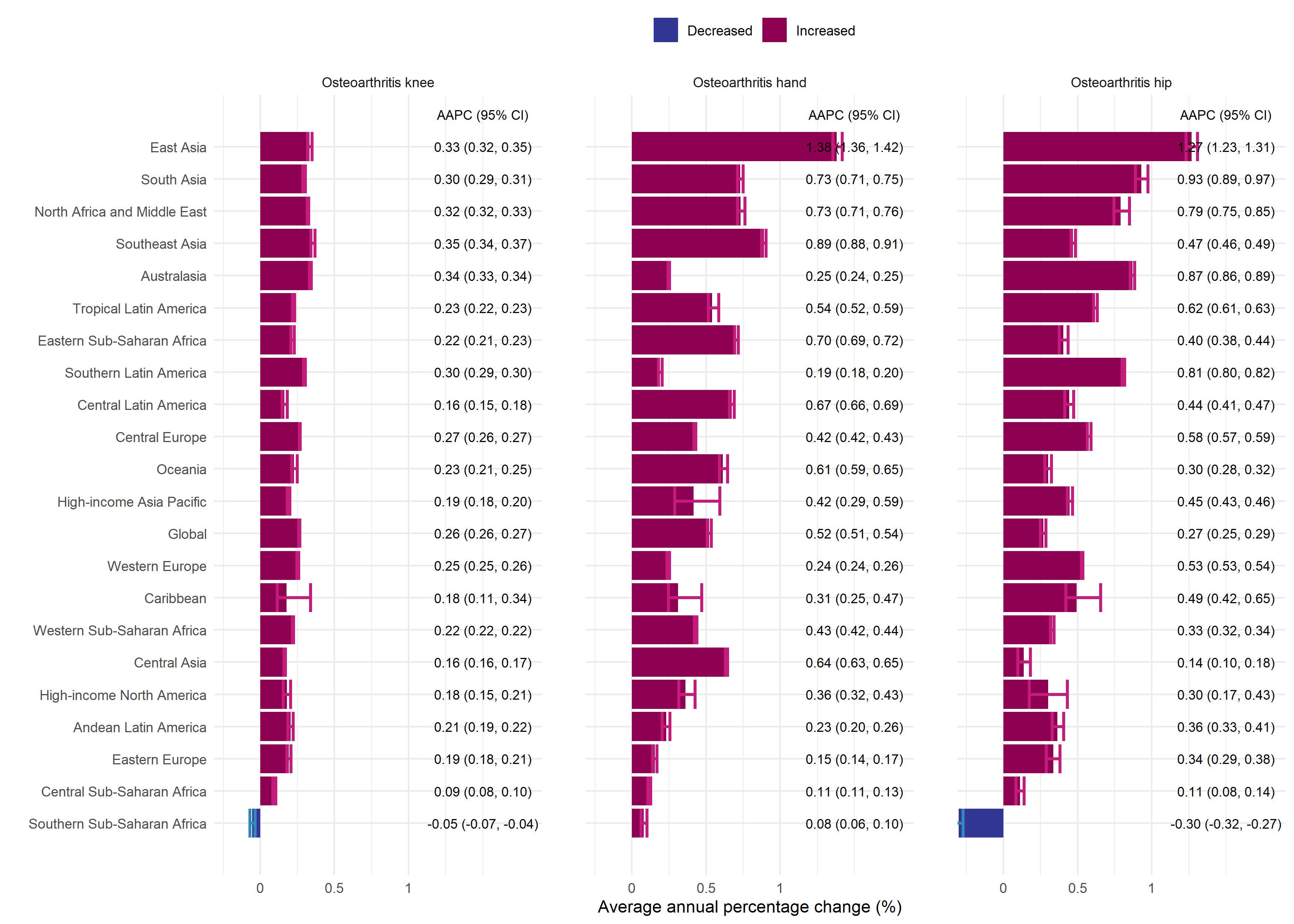


Table S1. Details of risk factors included in spatial heterogeneity analysis.

|  | Sources | Definitions | Index year of measurement |
| --- | --- | --- | --- |
| Demographic factors |  |  |  |
| Population size | GBD 2021: https://vizhub.healthdata.org/gbd-results/ |  | 1990 and 2021 |
| Proportion of adults aged ≥65 years | GBD 2021: https://vizhub.healthdata.org/gbd-results/ | Proportion of adults aged ≥65 to total population | 1990 and 2021 |
| Proportion of people living in urban area | World Bank:  https://databank.worldbank.org/source/world-development-indicators | Proportion of people employed in agriculture to total population | 1990 and 2021 |
| Socioeconomic factors |  |  |  |
| Proportion of people with upper secondary education or higher | World Bank:  https://databank.worldbank.org/source/world-development-indicators | Educational attainment at least completed upper secondary in population aged 25+ (%) | 2010 and 2021 |
| SDI | GBD 2021:  https://ghdx.healthdata.org/record/global-burden-disease-study-2021-gbd-2021-socio-demographic-index-sdi-1950%E2%80%932021 |  | 1990 and 2021 |
| UHC index | World Health Organization:  https://www.who.int/data/gho/data/indicators |  | 2000 and 2021 |
| Health indicators |  |  |  |
| Life expectancy | World Health Organization:  https://www.who.int/data/gho/data/indicators |  | 2000 and 2021 |
| Healthy life expectancy | World Health Organization:  https://www.who.int/data/gho/data/indicators |  | 2000 and 2021 |
| Health expenditure per capita | World Health Organization:  https://www.who.int/data/gho/data/indicators |  | 2000 and 2021 |
| Health expenditure as a percentage of GDP | World Health Organization:  https://www.who.int/data/gho/data/indicators |  | 2000 and 2021 |
| OA-related risk factors |  |  |  |
| Overweight | GBD 2021: https://vizhub.healthdata.org/gbd-results/ |  | 1990 and 2021 |
| Physical inactivity | GBD 2021: https://vizhub.healthdata.org/gbd-results/ |  | 1990 and 2021 |
| Proportion of people employed in agriculture | International Labour Organization:  https://ilostat.ilo.org/data/# | Proportion of people employed in agriculture to total employment | 1990 and 2021 |

GBD: Global burden of disease; GDP: gross domestic product; OA: osteoarthritis; SDI: socio-demographic index; UHC: universal health coverage.

Table S2. National expected lifetime risk estimates (%) of osteoarthritis and its subtypes for both sexes combined in 2021.

|  | OA | OA knee | OA hand | OA hip |
| --- | --- | --- | --- | --- |
| Afghanistan | 10.08 (9.92, 10.25) | 7.20 (7.08, 7.32) | 1.63 (1.54, 1.71) | 0.44 (0.37, 0.51) |
| Albania | 12.96 (12.65, 13.27) | 7.60 (7.41, 7.80) | 3.04 (2.87, 3.21) | 1.56 (1.36, 1.76) |
| Algeria | 13.00 (12.88, 13.13) | 8.64 (8.55, 8.73) | 2.84 (2.77, 2.91) | 0.67 (0.60, 0.74) |
| American Samoa | 14.67 (11.47, 17.88) | 9.61 (7.26, 11.95) | 3.52 (1.55, 5.50) | 0.80 (0.00, 2.17) |
| Andorra | 14.90 (13.62, 16.18) | 9.97 (8.93, 11.01) | 3.15 (2.42, 3.88) | 1.00 (0.71, 1.29) |
| Angola | 12.42 (12.22, 12.62) | 7.51 (7.37, 7.65) | 3.56 (3.44, 3.69) | 0.72 (0.62, 0.83) |
| Antigua and Barbuda | 15.01 (13.01, 17.01) | 9.88 (8.44, 11.31) | 3.70 (2.43, 4.98) | 0.75 (0.00, 1.58) |
| Argentina | 15.22 (15.16, 15.29) | 10.10 (10.05, 10.16) | 3.62 (3.58, 3.66) | 0.83 (0.81, 0.84) |
| Armenia | 13.69 (13.37, 14.01) | 6.86 (6.66, 7.06) | 4.57 (4.39, 4.74) | 1.60 (1.39, 1.82) |
| Australia | 16.18 (16.10, 16.26) | 10.83 (10.76, 10.89) | 3.67 (3.62, 3.72) | 0.97 (0.95, 0.99) |
| Austria | 14.79 (14.67, 14.91) | 9.93 (9.83, 10.02) | 3.13 (3.06, 3.20) | 0.97 (0.94, 1.00) |
| Azerbaijan | 13.40 (13.19, 13.61) | 6.74 (6.60, 6.87) | 4.54 (4.43, 4.66) | 1.47 (1.33, 1.61) |
| Bahamas | 14.84 (14.00, 15.68) | 9.85 (9.21, 10.49) | 3.58 (3.08, 4.08) | 0.75 (0.42, 1.08) |
| Bahrain | 13.24 (11.78, 14.70) | 8.75 (7.82, 9.67) | 2.88 (1.98, 3.78) | 0.73 (0.00, 1.56) |
| Bangladesh | 12.17 (12.11, 12.23) | 8.17 (8.13, 8.21) | 2.76 (2.72, 2.80) | 0.43 (0.41, 0.45) |
| Barbados | 15.49 (14.69, 16.28) | 10.25 (9.66, 10.85) | 3.73 (3.25, 4.20) | 0.82 (0.50, 1.14) |
| Belarus | 14.46 (14.32, 14.59) | 7.93 (7.84, 8.02) | 4.49 (4.41, 4.56) | 1.47 (1.39, 1.55) |
| Belgium | 14.77 (14.67, 14.88) | 9.87 (9.78, 9.95) | 3.20 (3.14, 3.26) | 0.95 (0.93, 0.97) |
| Belize | 15.00 (13.95, 16.05) | 10.13 (9.34, 10.93) | 3.31 (2.69, 3.92) | 0.83 (0.40, 1.26) |
| Benin | 12.59 (12.33, 12.85) | 8.37 (8.19, 8.55) | 2.68 (2.52, 2.83) | 0.79 (0.66, 0.91) |
| Bermuda | 16.03 (14.57, 17.49) | 10.61 (9.49, 11.74) | 3.82 (2.95, 4.69) | 0.90 (0.34, 1.45) |
| Bhutan | 12.61 (11.83, 13.40) | 8.70 (8.12, 9.28) | 2.54 (2.06, 3.01) | 0.51 (0.26, 0.76) |
| Bolivia (Plurinational State of) | 13.62 (13.46, 13.79) | 9.39 (9.26, 9.52) | 2.94 (2.84, 3.03) | 0.62 (0.56, 0.69) |
| Bosnia and Herzegovina | 13.72 (13.47, 13.98) | 7.63 (7.47, 7.79) | 3.80 (3.66, 3.94) | 1.62 (1.45, 1.79) |
| Botswana | 12.60 (12.18, 13.01) | 7.96 (7.66, 8.26) | 3.15 (2.90, 3.39) | 0.83 (0.64, 1.03) |
| Brazil | 15.75 (15.72, 15.79) | 10.09 (10.06, 10.12) | 4.16 (4.13, 4.18) | 0.86 (0.85, 0.88) |
| Brunei Darussalam | 17.22 (14.47, 19.98) | 12.07 (10.27, 13.87) | 3.99 (1.80, 6.19) | 0.63 (0.30, 0.97) |
| Bulgaria | 13.43 (13.28, 13.59) | 7.41 (7.31, 7.51) | 3.99 (3.91, 4.08) | 1.39 (1.30, 1.49) |
| Burkina Faso | 11.22 (11.02, 11.41) | 7.97 (7.83, 8.11) | 1.74 (1.62, 1.85) | 0.70 (0.61, 0.79) |
| Burundi | 10.73 (10.43, 11.04) | 7.25 (7.04, 7.46) | 2.01 (1.83, 2.19) | 0.70 (0.55, 0.85) |
| Cabo Verde | 13.40 (12.62, 14.19) | 8.80 (8.22, 9.39) | 2.93 (2.48, 3.38) | 0.89 (0.55, 1.23) |
| Cambodia | 10.63 (10.41, 10.84) | 7.01 (6.85, 7.16) | 2.32 (2.19, 2.45) | 0.47 (0.39, 0.55) |
| Cameroon | 12.29 (12.12, 12.47) | 8.37 (8.25, 8.49) | 2.37 (2.26, 2.47) | 0.79 (0.70, 0.88) |
| Canada | 12.51 (12.45, 12.57) | 6.98 (6.93, 7.02) | 3.87 (3.83, 3.91) | 0.78 (0.77, 0.79) |
| Central African Republic | 10.02 (9.56, 10.48) | 6.72 (6.41, 7.03) | 2.06 (1.78, 2.34) | 0.57 (0.34, 0.80) |
| Chad | 10.84 (10.57, 11.10) | 7.57 (7.38, 7.75) | 1.83 (1.68, 1.99) | 0.64 (0.52, 0.77) |
| Chile | 15.54 (15.44, 15.65) | 10.46 (10.38, 10.55) | 3.48 (3.42, 3.55) | 0.89 (0.87, 0.91) |
| China | 14.97 (14.95, 14.98) | 10.49 (10.48, 10.50) | 3.15 (3.14, 3.16) | 0.57 (0.57, 0.58) |
| Colombia | 14.91 (14.84, 14.98) | 9.99 (9.94, 10.05) | 3.49 (3.45, 3.53) | 0.69 (0.67, 0.72) |
| Comoros | 12.03 (11.12, 12.93) | 7.90 (7.28, 8.52) | 2.54 (2.01, 3.07) | 0.83 (0.38, 1.28) |
| Congo | 12.33 (11.89, 12.77) | 7.73 (7.43, 8.03) | 3.14 (2.87, 3.41) | 0.79 (0.55, 1.03) |
| Cook Islands | 15.28 (11.34, 19.22) | 9.97 (7.05, 12.90) | 3.69 (1.26, 6.11) | 0.85 (0.00, 2.46) |
| Costa Rica | 15.18 (14.96, 15.41) | 10.23 (10.05, 10.40) | 3.47 (3.34, 3.61) | 0.74 (0.66, 0.82) |
| Croatia | 14.34 (14.12, 14.56) | 7.86 (7.72, 8.00) | 4.10 (3.98, 4.23) | 1.71 (1.56, 1.86) |
| Cuba | 14.59 (14.48, 14.69) | 9.88 (9.79, 9.96) | 3.24 (3.18, 3.30) | 0.75 (0.72, 0.79) |
| Cyprus | 14.49 (13.71, 15.27) | 9.69 (9.17, 10.22) | 3.13 (2.55, 3.72) | 0.91 (0.81, 1.01) |
| Czechia | 14.71 (14.58, 14.84) | 7.93 (7.85, 8.01) | 4.34 (4.27, 4.41) | 1.80 (1.72, 1.88) |
| CÃ´te d'Ivoire | 12.14 (11.93, 12.35) | 8.16 (8.02, 8.30) | 2.45 (2.32, 2.57) | 0.76 (0.66, 0.87) |
| Democratic People's Republic of Korea | 13.43 (13.30, 13.57) | 9.73 (9.63, 9.83) | 2.50 (2.42, 2.59) | 0.44 (0.39, 0.48) |
| Democratic Republic of the Congo | 11.27 (11.17, 11.38) | 7.39 (7.31, 7.46) | 2.47 (2.40, 2.53) | 0.70 (0.64, 0.75) |
| Denmark | 14.58 (14.43, 14.73) | 9.67 (9.55, 9.79) | 3.12 (3.04, 3.21) | 1.03 (1.00, 1.07) |
| Djibouti | 12.06 (11.02, 13.09) | 7.72 (7.03, 8.41) | 2.79 (2.16, 3.43) | 0.80 (0.26, 1.35) |
| Dominica | 14.35 (12.54, 16.17) | 9.70 (8.33, 11.08) | 3.22 (2.12, 4.32) | 0.73 (0.03, 1.42) |
| Dominican Republic | 14.95 (14.76, 15.13) | 9.95 (9.82, 10.09) | 3.48 (3.37, 3.59) | 0.79 (0.71, 0.86) |
| Ecuador | 15.27 (15.12, 15.42) | 10.36 (10.25, 10.47) | 3.38 (3.29, 3.48) | 0.81 (0.75, 0.88) |
| Egypt | 12.14 (12.06, 12.21) | 8.36 (8.30, 8.41) | 2.31 (2.27, 2.34) | 0.62 (0.59, 0.66) |
| El Salvador | 14.75 (14.56, 14.94) | 9.99 (9.84, 10.14) | 3.36 (3.25, 3.47) | 0.68 (0.62, 0.75) |
| Equatorial Guinea | 12.82 (11.93, 13.71) | 8.08 (7.47, 8.69) | 3.22 (2.68, 3.75) | 0.87 (0.41, 1.33) |
| Eritrea | 10.82 (10.29, 11.34) | 7.20 (6.86, 7.54) | 2.22 (1.89, 2.55) | 0.65 (0.38, 0.92) |
| Estonia | 15.56 (15.20, 15.92) | 8.39 (8.15, 8.64) | 4.84 (4.64, 5.05) | 1.77 (1.54, 1.99) |
| Eswatini | 11.33 (10.68, 11.97) | 7.40 (6.94, 7.86) | 2.58 (2.19, 2.97) | 0.71 (0.39, 1.04) |
| Ethiopia | 12.96 (12.86, 13.06) | 7.67 (7.60, 7.74) | 3.80 (3.74, 3.86) | 0.84 (0.79, 0.89) |
| Fiji | 13.47 (12.60, 14.34) | 8.89 (8.26, 9.51) | 3.24 (2.66, 3.81) | 0.63 (0.27, 0.99) |
| Finland | 14.87 (14.72, 15.01) | 9.97 (9.85, 10.09) | 3.14 (3.06, 3.22) | 0.99 (0.96, 1.03) |
| France | 14.83 (14.78, 14.87) | 9.86 (9.83, 9.90) | 3.20 (3.18, 3.23) | 1.01 (1.00, 1.02) |
| Gabon | 12.85 (12.26, 13.44) | 8.22 (7.80, 8.63) | 3.04 (2.69, 3.40) | 0.90 (0.60, 1.20) |
| Gambia | 12.23 (11.64, 12.82) | 8.05 (7.63, 8.46) | 2.75 (2.39, 3.10) | 0.72 (0.44, 1.00) |
| Georgia | 12.95 (12.76, 13.14) | 6.70 (6.57, 6.83) | 4.10 (3.99, 4.21) | 1.46 (1.35, 1.56) |
| Germany | 14.85 (14.81, 14.89) | 9.94 (9.91, 9.97) | 3.17 (3.15, 3.20) | 0.99 (0.98, 1.00) |
| Ghana | 12.89 (12.73, 13.06) | 8.41 (8.29, 8.52) | 3.00 (2.90, 3.10) | 0.78 (0.70, 0.86) |
| Greece | 14.47 (14.37, 14.58) | 9.91 (9.83, 10.00) | 2.89 (2.83, 2.94) | 0.89 (0.87, 0.92) |
| Greenland | 12.29 (9.43, 15.15) | 7.09 (5.09, 9.09) | 3.64 (1.64, 5.63) | 0.78 (0.37, 1.19) |
| Grenada | 14.15 (12.50, 15.80) | 9.46 (8.24, 10.69) | 3.34 (2.31, 4.37) | 0.67 (0.06, 1.29) |
| Guam | 15.29 (14.15, 16.43) | 9.86 (8.96, 10.76) | 3.82 (3.16, 4.48) | 0.85 (0.44, 1.25) |
| Guatemala | 13.58 (13.35, 13.81) | 9.38 (9.22, 9.54) | 2.87 (2.72, 3.02) | 0.59 (0.49, 0.68) |
| Guinea | 11.22 (11.01, 11.43) | 7.81 (7.65, 7.96) | 1.95 (1.83, 2.07) | 0.67 (0.58, 0.77) |
| Guinea-Bissau | 10.87 (10.10, 11.65) | 7.49 (6.96, 8.02) | 2.06 (1.59, 2.53) | 0.60 (0.23, 0.97) |
| Guyana | 13.60 (12.96, 14.24) | 9.18 (8.69, 9.67) | 3.12 (2.75, 3.49) | 0.64 (0.39, 0.89) |
| Haiti | 11.92 (11.70, 12.13) | 8.38 (8.22, 8.54) | 2.32 (2.20, 2.45) | 0.51 (0.42, 0.59) |
| Honduras | 13.39 (13.14, 13.63) | 9.29 (9.11, 9.47) | 2.80 (2.65, 2.95) | 0.57 (0.47, 0.66) |
| Hungary | 14.55 (14.42, 14.68) | 7.86 (7.78, 7.95) | 4.32 (4.25, 4.39) | 1.74 (1.66, 1.82) |
| Iceland | 15.24 (14.57, 15.90) | 10.13 (9.59, 10.67) | 3.25 (2.87, 3.63) | 1.11 (0.95, 1.27) |
| India | 13.23 (13.21, 13.25) | 8.60 (8.59, 8.62) | 3.40 (3.39, 3.41) | 0.48 (0.47, 0.48) |
| Indonesia | 12.02 (11.97, 12.07) | 7.23 (7.19, 7.27) | 3.42 (3.38, 3.45) | 0.58 (0.56, 0.60) |
| Iran (Islamic Republic of) | 13.49 (13.42, 13.56) | 8.52 (8.47, 8.57) | 3.39 (3.35, 3.43) | 0.77 (0.74, 0.81) |
| Iraq | 12.61 (12.48, 12.74) | 8.44 (8.35, 8.53) | 2.65 (2.58, 2.72) | 0.67 (0.61, 0.73) |
| Ireland | 14.91 (14.72, 15.11) | 10.02 (9.86, 10.17) | 3.14 (3.03, 3.26) | 0.99 (0.95, 1.03) |
| Israel | 14.91 (14.76, 15.06) | 10.02 (9.90, 10.14) | 3.20 (3.12, 3.29) | 0.93 (0.89, 0.96) |
| Italy | 15.48 (15.43, 15.52) | 9.99 (9.96, 10.03) | 3.85 (3.82, 3.88) | 0.91 (0.90, 0.92) |
| Jamaica | 14.71 (14.45, 14.97) | 9.91 (9.70, 10.12) | 3.31 (3.16, 3.45) | 0.77 (0.68, 0.86) |
| Japan | 18.01 (17.98, 18.04) | 12.07 (12.05, 12.10) | 4.83 (4.81, 4.84) | 0.61 (0.60, 0.62) |
| Jordan | 13.70 (13.42, 13.99) | 9.00 (8.79, 9.20) | 3.02 (2.87, 3.18) | 0.81 (0.66, 0.96) |
| Kazakhstan | 13.81 (13.64, 13.97) | 6.76 (6.66, 6.87) | 5.00 (4.91, 5.09) | 1.44 (1.32, 1.56) |
| Kenya | 13.11 (12.97, 13.25) | 7.88 (7.78, 7.97) | 3.71 (3.62, 3.80) | 0.88 (0.81, 0.94) |
| Kiribati | 13.23 (10.00, 16.45) | 8.75 (6.45, 11.05) | 3.18 (1.10, 5.27) | 0.59 (0.00, 1.92) |
| Kuwait | 14.51 (14.10, 14.92) | 9.48 (9.18, 9.78) | 3.17 (2.95, 3.39) | 0.95 (0.75, 1.15) |
| Kyrgyzstan | 13.31 (12.96, 13.66) | 6.58 (6.36, 6.80) | 4.61 (4.42, 4.80) | 1.46 (1.22, 1.70) |
| Lao People's Democratic Republic | 10.80 (10.48, 11.11) | 7.05 (6.82, 7.28) | 2.38 (2.19, 2.57) | 0.51 (0.39, 0.62) |
| Latvia | 15.02 (14.74, 15.31) | 8.18 (7.99, 8.37) | 4.63 (4.47, 4.79) | 1.65 (1.48, 1.82) |
| Lebanon | 13.55 (13.35, 13.75) | 8.78 (8.63, 8.93) | 3.15 (3.04, 3.26) | 0.75 (0.67, 0.84) |
| Lesotho | 10.43 (10.06, 10.80) | 6.77 (6.50, 7.04) | 2.49 (2.28, 2.70) | 0.59 (0.43, 0.74) |
| Liberia | 12.30 (11.88, 12.72) | 8.30 (8.00, 8.60) | 2.44 (2.20, 2.68) | 0.79 (0.59, 0.99) |
| Libya | 13.20 (12.95, 13.45) | 8.68 (8.49, 8.86) | 2.94 (2.80, 3.08) | 0.74 (0.62, 0.85) |
| Lithuania | 15.00 (14.75, 15.24) | 8.17 (8.01, 8.33) | 4.62 (4.48, 4.76) | 1.64 (1.49, 1.79) |
| Luxembourg | 14.86 (14.36, 15.36) | 9.91 (9.51, 10.31) | 3.20 (2.90, 3.50) | 0.98 (0.87, 1.09) |
| Madagascar | 10.59 (10.38, 10.80) | 7.30 (7.16, 7.44) | 1.81 (1.69, 1.93) | 0.69 (0.59, 0.79) |
| Malawi | 10.74 (10.55, 10.92) | 7.30 (7.16, 7.43) | 2.04 (1.94, 2.15) | 0.68 (0.59, 0.76) |
| Malaysia | 12.42 (12.30, 12.54) | 7.65 (7.56, 7.74) | 3.31 (3.24, 3.38) | 0.63 (0.58, 0.67) |
| Maldives | 12.69 (11.46, 13.93) | 7.62 (6.71, 8.52) | 3.56 (2.82, 4.31) | 0.65 (0.17, 1.13) |
| Mali | 11.22 (10.98, 11.46) | 7.71 (7.55, 7.88) | 2.09 (1.94, 2.23) | 0.66 (0.53, 0.78) |
| Malta | 14.91 (14.37, 15.45) | 9.94 (9.51, 10.37) | 3.22 (2.90, 3.54) | 0.98 (0.86, 1.10) |
| Marshall Islands | 13.03 (7.60, 18.47) | 8.37 (4.46, 12.27) | 3.40 (0.02, 6.78) | 0.58 (0.00, 2.88) |
| Mauritania | 13.05 (12.63, 13.46) | 8.76 (8.47, 9.05) | 2.62 (2.38, 2.85) | 0.88 (0.68, 1.08) |
| Mauritius | 12.86 (12.45, 13.27) | 7.81 (7.50, 8.12) | 3.61 (3.36, 3.85) | 0.64 (0.49, 0.78) |
| Mexico | 15.75 (15.70, 15.81) | 10.11 (10.08, 10.15) | 4.25 (4.21, 4.28) | 0.77 (0.75, 0.79) |
| Micronesia (Federated States of) | 13.53 (10.76, 16.31) | 8.88 (6.85, 10.91) | 3.29 (1.57, 5.02) | 0.64 (0.00, 1.78) |
| Monaco | 14.94 (13.38, 16.50) | 10.04 (8.76, 11.33) | 3.12 (2.25, 4.00) | 1.04 (0.64, 1.44) |
| Mongolia | 13.46 (12.69, 14.24) | 6.47 (6.03, 6.91) | 5.00 (4.59, 5.42) | 1.39 (0.81, 1.97) |
| Montenegro | 13.49 (12.94, 14.04) | 7.52 (7.15, 7.89) | 3.97 (3.67, 4.27) | 1.35 (1.01, 1.70) |
| Morocco | 12.61 (12.50, 12.72) | 8.43 (8.35, 8.51) | 2.64 (2.58, 2.71) | 0.67 (0.62, 0.73) |
| Mozambique | 10.48 (10.32, 10.63) | 7.13 (7.01, 7.24) | 1.98 (1.89, 2.08) | 0.65 (0.58, 0.72) |
| Myanmar | 11.24 (11.15, 11.32) | 7.14 (7.08, 7.20) | 2.79 (2.74, 2.84) | 0.50 (0.47, 0.53) |
| Namibia | 11.70 (11.28, 12.12) | 7.58 (7.28, 7.89) | 2.70 (2.45, 2.95) | 0.73 (0.53, 0.92) |
| Nauru | 13.26 (2.97, 23.55) | 8.72 (1.26, 16.18) | 3.23 (0.00, 9.64) | 0.61 (0.00, 4.99) |
| Nepal | 11.59 (11.47, 11.70) | 8.16 (8.08, 8.25) | 2.16 (2.08, 2.23) | 0.44 (0.40, 0.48) |
| Netherlands | 15.05 (14.96, 15.14) | 10.18 (10.11, 10.25) | 3.15 (3.10, 3.21) | 0.98 (0.96, 1.00) |
| New Zealand | 16.65 (16.45, 16.85) | 10.74 (10.58, 10.89) | 4.33 (4.19, 4.46) | 0.94 (0.90, 0.98) |
| Nicaragua | 14.24 (13.93, 14.55) | 9.78 (9.56, 10.00) | 3.05 (2.85, 3.25) | 0.65 (0.53, 0.78) |
| Niger | 11.14 (10.89, 11.39) | 7.80 (7.63, 7.97) | 1.86 (1.71, 2.01) | 0.67 (0.55, 0.79) |
| Nigeria | 13.18 (13.11, 13.25) | 8.46 (8.41, 8.51) | 3.21 (3.16, 3.25) | 0.79 (0.76, 0.82) |
| Niue | 14.53 (2.22, 26.85) | 9.49 (0.25, 18.74) | 3.55 (0.00, 11.13) | 0.74 (0.00, 5.56) |
| North Macedonia | 12.88 (12.19, 13.58) | 7.33 (6.95, 7.71) | 3.59 (3.21, 3.98) | 1.28 (0.75, 1.82) |
| Northern Mariana Islands | 14.32 (10.73, 17.90) | 9.33 (6.72, 11.94) | 3.50 (1.27, 5.73) | 0.75 (0.00, 2.26) |
| Norway | 15.60 (15.44, 15.77) | 9.94 (9.81, 10.07) | 3.99 (3.89, 4.10) | 0.96 (0.93, 1.00) |
| Oman | 13.20 (12.62, 13.78) | 8.65 (8.26, 9.04) | 2.94 (2.59, 3.29) | 0.74 (0.44, 1.03) |
| Pakistan | 12.01 (11.95, 12.07) | 7.45 (7.41, 7.49) | 3.28 (3.24, 3.31) | 0.49 (0.47, 0.51) |
| Palau | 14.14 (8.31, 19.96) | 9.25 (5.02, 13.48) | 3.39 (0.00, 6.88) | 0.74 (0.00, 3.38) |
| Palestine | 12.89 (12.43, 13.35) | 8.44 (8.12, 8.76) | 2.95 (2.67, 3.23) | 0.66 (0.44, 0.88) |
| Panama | 14.95 (14.69, 15.20) | 10.03 (9.83, 10.22) | 3.45 (3.30, 3.60) | 0.71 (0.62, 0.81) |
| Papua New Guinea | 11.45 (11.15, 11.74) | 7.94 (7.71, 8.16) | 2.21 (2.03, 2.39) | 0.51 (0.40, 0.63) |
| Paraguay | 14.33 (14.12, 14.54) | 9.66 (9.50, 9.82) | 3.23 (3.11, 3.35) | 0.72 (0.64, 0.80) |
| Peru | 14.73 (14.65, 14.81) | 9.89 (9.83, 9.96) | 3.43 (3.38, 3.47) | 0.73 (0.70, 0.75) |
| Philippines | 11.73 (11.66, 11.80) | 6.75 (6.70, 6.80) | 3.60 (3.56, 3.65) | 0.57 (0.55, 0.60) |
| Poland | 15.42 (15.35, 15.49) | 8.00 (7.95, 8.05) | 4.95 (4.90, 4.99) | 1.90 (1.86, 1.95) |
| Portugal | 14.72 (14.62, 14.83) | 9.95 (9.86, 10.03) | 3.08 (3.02, 3.14) | 0.93 (0.91, 0.96) |
| Puerto Rico | 16.43 (16.24, 16.63) | 10.90 (10.75, 11.05) | 3.85 (3.74, 3.96) | 0.97 (0.90, 1.04) |
| Qatar | 13.96 (12.55, 15.37) | 9.21 (8.30, 10.13) | 2.91 (2.04, 3.77) | 0.90 (0.11, 1.68) |
| Republic of Korea | 17.98 (17.91, 18.04) | 12.84 (12.80, 12.89) | 3.98 (3.93, 4.02) | 0.64 (0.63, 0.65) |
| Republic of Moldova | 14.22 (13.99, 14.44) | 8.04 (7.89, 8.20) | 3.92 (3.79, 4.04) | 1.63 (1.49, 1.77) |
| Romania | 13.56 (13.47, 13.65) | 7.66 (7.60, 7.72) | 3.58 (3.52, 3.63) | 1.63 (1.57, 1.69) |
| Russian Federation | 15.47 (15.43, 15.51) | 8.03 (8.01, 8.06) | 5.30 (5.27, 5.32) | 1.66 (1.64, 1.68) |
| Rwanda | 11.24 (10.97, 11.51) | 7.71 (7.52, 7.89) | 1.98 (1.82, 2.14) | 0.76 (0.63, 0.90) |
| Saint Kitts and Nevis | 14.94 (12.64, 17.24) | 9.97 (8.25, 11.68) | 3.52 (2.12, 4.92) | 0.77 (0.00, 1.69) |
| Saint Lucia | 14.68 (13.59, 15.77) | 9.88 (9.05, 10.70) | 3.35 (2.69, 4.00) | 0.75 (0.32, 1.18) |
| Saint Vincent and the Grenadines | 14.41 (12.76, 16.06) | 9.73 (8.54, 10.92) | 3.23 (2.19, 4.27) | 0.73 (0.04, 1.43) |
| Samoa | 13.96 (12.25, 15.68) | 9.18 (7.91, 10.44) | 3.32 (2.27, 4.38) | 0.70 (0.01, 1.40) |
| San Marino | 15.26 (13.44, 17.09) | 10.24 (8.73, 11.75) | 3.23 (2.22, 4.23) | 1.02 (0.57, 1.47) |
| Sao Tome and Principe | 13.48 (11.45, 15.51) | 8.59 (7.19, 10.00) | 3.34 (2.10, 4.57) | 0.84 (0.00, 1.86) |
| Saudi Arabia | 13.57 (13.35, 13.78) | 8.93 (8.78, 9.08) | 2.94 (2.81, 3.06) | 0.82 (0.71, 0.94) |
| Senegal | 12.19 (11.99, 12.39) | 8.19 (8.05, 8.34) | 2.50 (2.38, 2.62) | 0.74 (0.65, 0.83) |
| Serbia | 13.66 (13.53, 13.79) | 7.68 (7.59, 7.77) | 3.72 (3.65, 3.79) | 1.57 (1.50, 1.65) |
| Seychelles | 12.81 (11.05, 14.57) | 7.77 (6.47, 9.07) | 3.60 (2.52, 4.67) | 0.64 (0.00, 1.28) |
| Sierra Leone | 11.79 (11.46, 12.12) | 7.96 (7.73, 8.18) | 2.35 (2.15, 2.54) | 0.72 (0.56, 0.88) |
| Singapore | 17.81 (17.61, 18.02) | 12.46 (12.30, 12.62) | 4.15 (4.02, 4.28) | 0.65 (0.62, 0.69) |
| Slovakia | 14.40 (14.21, 14.58) | 7.79 (7.66, 7.91) | 4.30 (4.20, 4.40) | 1.67 (1.55, 1.79) |
| Slovenia | 14.97 (14.69, 15.24) | 8.06 (7.88, 8.25) | 4.35 (4.19, 4.51) | 1.90 (1.72, 2.07) |
| Solomon Islands | 12.51 (11.05, 13.97) | 8.36 (7.32, 9.40) | 2.83 (1.92, 3.74) | 0.57 (0.00, 1.20) |
| Somalia | 9.70 (9.43, 9.98) | 6.72 (6.53, 6.90) | 1.74 (1.57, 1.90) | 0.55 (0.41, 0.69) |
| South Africa | 14.10 (14.02, 14.19) | 8.39 (8.33, 8.45) | 4.12 (4.06, 4.17) | 0.99 (0.95, 1.04) |
| South Sudan | 10.55 (10.28, 10.82) | 7.35 (7.15, 7.54) | 1.72 (1.58, 1.87) | 0.72 (0.59, 0.84) |
| Spain | 14.91 (14.85, 14.96) | 9.96 (9.92, 10.00) | 3.15 (3.13, 3.18) | 1.02 (1.01, 1.03) |
| Sri Lanka | 12.17 (12.04, 12.31) | 7.58 (7.48, 7.68) | 3.17 (3.08, 3.25) | 0.58 (0.53, 0.63) |
| Sudan | 11.88 (11.75, 12.01) | 8.11 (8.01, 8.20) | 2.28 (2.21, 2.35) | 0.62 (0.56, 0.68) |
| Suriname | 14.90 (14.23, 15.56) | 9.86 (9.35, 10.37) | 3.59 (3.19, 3.98) | 0.76 (0.50, 1.02) |
| Sweden | 14.11 (13.99, 14.22) | 8.79 (8.70, 8.88) | 3.55 (3.48, 3.62) | 0.94 (0.92, 0.97) |
| Switzerland | 14.72 (14.60, 14.84) | 9.83 (9.74, 9.93) | 3.19 (3.13, 3.26) | 0.92 (0.89, 0.94) |
| Syrian Arab Republic | 12.64 (12.42, 12.86) | 8.46 (8.31, 8.61) | 2.68 (2.56, 2.79) | 0.66 (0.54, 0.77) |
| Taiwan (Province of China) | 15.60 (15.51, 15.69) | 10.79 (10.72, 10.86) | 3.48 (3.43, 3.53) | 0.59 (0.56, 0.62) |
| Tajikistan | 12.02 (11.69, 12.35) | 6.17 (5.96, 6.37) | 3.84 (3.66, 4.01) | 1.30 (1.08, 1.53) |
| Thailand | 12.74 (12.68, 12.79) | 8.30 (8.26, 8.34) | 2.91 (2.88, 2.94) | 0.66 (0.64, 0.67) |
| Timor-Leste | 10.72 (9.95, 11.48) | 6.76 (6.21, 7.31) | 2.66 (2.20, 3.13) | 0.47 (0.19, 0.75) |
| Togo | 11.98 (11.60, 12.36) | 8.02 (7.76, 8.28) | 2.52 (2.28, 2.75) | 0.70 (0.52, 0.89) |
| Tokelau | 14.23 (0.17, 28.30) | 9.32 (0.00, 20.08) | 3.43 (0.00, 11.69) | 0.72 (0.00, 6.11) |
| Tonga | 14.13 (12.11, 16.15) | 9.44 (7.93, 10.96) | 3.17 (1.94, 4.40) | 0.74 (0.00, 1.51) |
| Trinidad and Tobago | 14.87 (14.50, 15.24) | 9.92 (9.63, 10.20) | 3.49 (3.27, 3.71) | 0.78 (0.64, 0.92) |
| Tunisia | 13.10 (12.94, 13.25) | 8.56 (8.45, 8.68) | 3.01 (2.92, 3.10) | 0.70 (0.63, 0.77) |
| Turkmenistan | 13.17 (12.90, 13.45) | 6.61 (6.42, 6.80) | 4.47 (4.32, 4.62) | 1.45 (1.29, 1.62) |
| Tuvalu | 13.60 (6.85, 20.35) | 8.89 (3.95, 13.83) | 3.33 (0.00, 7.52) | 0.64 (0.00, 3.37) |
| TÃ¼rkiye | 13.54 (13.48, 13.60) | 9.10 (9.06, 9.15) | 2.80 (2.77, 2.84) | 0.76 (0.74, 0.79) |
| Uganda | 11.14 (10.99, 11.29) | 7.53 (7.43, 7.64) | 2.12 (2.03, 2.21) | 0.74 (0.67, 0.81) |
| Ukraine | 14.98 (14.92, 15.05) | 7.93 (7.89, 7.97) | 4.95 (4.91, 4.99) | 1.58 (1.54, 1.62) |
| United Arab Emirates | 13.06 (11.81, 14.32) | 8.79 (7.96, 9.62) | 2.51 (1.84, 3.17) | 0.83 (0.08, 1.58) |
| United Kingdom | 16.03 (15.99, 16.08) | 10.58 (10.54, 10.61) | 3.73 (3.70, 3.76) | 1.05 (1.04, 1.06) |
| United Republic of Tanzania | 11.72 (11.61, 11.84) | 7.81 (7.73, 7.89) | 2.33 (2.27, 2.40) | 0.81 (0.75, 0.87) |
| United States Virgin Islands | 15.98 (14.31, 17.65) | 10.62 (9.44, 11.80) | 3.73 (2.65, 4.82) | 0.92 (0.18, 1.66) |
| United States of America | 16.75 (16.72, 16.77) | 10.42 (10.40, 10.43) | 4.69 (4.68, 4.70) | 1.12 (1.12, 1.13) |
| Uruguay | 15.21 (15.01, 15.41) | 10.17 (10.01, 10.34) | 3.50 (3.38, 3.62) | 0.85 (0.81, 0.90) |
| Uzbekistan | 13.54 (13.39, 13.70) | 6.75 (6.65, 6.84) | 4.60 (4.52, 4.68) | 1.54 (1.43, 1.65) |
| Vanuatu | 11.95 (10.03, 13.87) | 8.43 (7.05, 9.81) | 2.14 (0.95, 3.33) | 0.57 (0.00, 1.36) |
| Venezuela (Bolivarian Republic of) | 14.46 (14.37, 14.55) | 9.74 (9.67, 9.81) | 3.36 (3.31, 3.41) | 0.65 (0.62, 0.68) |
| Viet Nam | 11.24 (11.18, 11.31) | 7.21 (7.16, 7.25) | 2.67 (2.63, 2.71) | 0.51 (0.49, 0.53) |
| Yemen | 11.09 (10.92, 11.25) | 7.52 (7.40, 7.63) | 2.23 (2.13, 2.32) | 0.51 (0.44, 0.58) |
| Zambia | 10.80 (10.61, 10.99) | 7.24 (7.10, 7.38) | 2.19 (2.08, 2.30) | 0.67 (0.58, 0.76) |
| Zimbabwe | 10.19 (10.02, 10.37) | 7.03 (6.90, 7.15) | 1.86 (1.77, 1.96) | 0.61 (0.53, 0.69) |

Table S3. Global and regional expected lifetime risks (%) of osteoarthritis and its subtypes for men in 2021.

|  | OA | OA knee | OA hand | OA hip |
| --- | --- | --- | --- | --- |
| Global | 11.94 (11.93, 11.95) | 7.89 (7.88, 7.90) | 2.43 (2.43, 2.44) | 0.71 (0.70, 0.71) |
| Low SDI | 10.05 (10.01, 10.09) | 7.07 (7.04, 7.10) | 1.50 (1.48, 1.52) | 0.59 (0.57, 0.61) |
| Low-middle SDI | 10.57 (10.55, 10.60) | 7.34 (7.33, 7.36) | 1.77 (1.76, 1.79) | 0.52 (0.51, 0.53) |
| Middle SDI | 11.80 (11.78, 11.81) | 7.74 (7.72, 7.75) | 2.48 (2.47, 2.49) | 0.66 (0.65, 0.66) |
| High-middle SDI | 12.27 (12.25, 12.29) | 7.78 (7.76, 7.79) | 2.66 (2.65, 2.67) | 0.93 (0.92, 0.94) |
| High SDI | 13.75 (13.73, 13.76) | 8.94 (8.93, 8.96) | 3.10 (3.09, 3.11) | 0.90 (0.89, 0.90) |
| Central Europe, Eastern Europe, and Central Asia | 12.77 (12.73, 12.80) | 7.16 (7.13, 7.18) | 3.03 (3.01, 3.05) | 1.81 (1.79, 1.84) |
| Central Asia | 10.63 (10.49, 10.77) | 6.09 (6.00, 6.17) | 2.11 (2.04, 2.17) | 1.55 (1.44, 1.65) |
| Central Europe | 12.58 (12.52, 12.65) | 7.13 (7.09, 7.17) | 2.83 (2.80, 2.86) | 1.81 (1.77, 1.85) |
| Eastern Europe | 13.50 (13.44, 13.55) | 7.49 (7.46, 7.53) | 3.44 (3.42, 3.47) | 1.87 (1.83, 1.91) |
| High-income | 13.74 (13.72, 13.76) | 9.07 (9.06, 9.08) | 2.97 (2.96, 2.98) | 0.90 (0.90, 0.91) |
| Australasia | 14.19 (14.08, 14.30) | 9.61 (9.52, 9.71) | 2.76 (2.70, 2.83) | 0.96 (0.93, 0.98) |
| High-income Asia Pacific | 14.72 (14.68, 14.75) | 10.04 (10.01, 10.08) | 3.27 (3.25, 3.29) | 0.65 (0.64, 0.66) |
| High-income North America | 14.27 (14.24, 14.30) | 8.94 (8.92, 8.97) | 3.61 (3.59, 3.63) | 1.01 (1.00, 1.02) |
| Southern Latin America | 13.21 (13.12, 13.29) | 8.95 (8.89, 9.02) | 2.59 (2.54, 2.63) | 0.85 (0.83, 0.87) |
| Western Europe | 12.82 (12.80, 12.85) | 8.63 (8.61, 8.66) | 2.37 (2.36, 2.38) | 0.93 (0.92, 0.94) |
| Latin America and Caribbean | 13.38 (13.35, 13.41) | 8.91 (8.89, 8.94) | 2.79 (2.77, 2.81) | 0.83 (0.81, 0.84) |
| Andean Latin America | 12.31 (12.22, 12.40) | 8.54 (8.47, 8.61) | 2.12 (2.08, 2.16) | 0.77 (0.74, 0.80) |
| Caribbean | 12.67 (12.57, 12.76) | 8.73 (8.66, 8.80) | 2.27 (2.22, 2.31) | 0.80 (0.76, 0.84) |
| Central Latin America | 13.44 (13.40, 13.49) | 8.95 (8.91, 8.98) | 2.88 (2.85, 2.90) | 0.77 (0.75, 0.79) |
| Tropical Latin America | 13.77 (13.72, 13.82) | 9.02 (8.98, 9.06) | 3.00 (2.97, 3.03) | 0.91 (0.89, 0.93) |
| North Africa and Middle East | 11.45 (11.41, 11.48) | 7.88 (7.86, 7.91) | 1.85 (1.83, 1.87) | 0.72 (0.70, 0.74) |
| South Asia | 10.55 (10.53, 10.57) | 7.42 (7.41, 7.44) | 1.77 (1.76, 1.79) | 0.40 (0.39, 0.41) |
| Southeast Asia, East Asia, and Oceania | 11.51 (11.49, 11.53) | 7.43 (7.41, 7.44) | 2.56 (2.55, 2.57) | 0.59 (0.58, 0.60) |
| East Asia | 11.99 (11.97, 12.01) | 7.94 (7.92, 7.96) | 2.53 (2.52, 2.55) | 0.58 (0.57, 0.59) |
| Oceania | 10.17 (9.86, 10.49) | 6.64 (6.40, 6.88) | 2.06 (1.90, 2.23) | 0.59 (0.46, 0.72) |
| Southeast Asia | 10.06 (10.02, 10.09) | 5.91 (5.89, 5.94) | 2.60 (2.58, 2.61) | 0.61 (0.60, 0.63) |
| Sub-Saharan Africa | 10.83 (10.79, 10.87) | 7.31 (7.28, 7.34) | 1.87 (1.85, 1.89) | 0.81 (0.79, 0.83) |
| Central Sub-Saharan Africa | 9.94 (9.82, 10.07) | 6.76 (6.67, 6.85) | 1.66 (1.60, 1.73) | 0.71 (0.64, 0.78) |
| Eastern Sub-Saharan Africa | 10.41 (10.34, 10.47) | 6.97 (6.93, 7.02) | 1.82 (1.79, 1.85) | 0.80 (0.76, 0.83) |
| Southern Sub-Saharan Africa | 12.28 (12.17, 12.39) | 8.02 (7.95, 8.10) | 2.35 (2.29, 2.41) | 1.12 (1.05, 1.18) |
| Western Sub-Saharan Africa | 11.03 (10.97, 11.09) | 7.57 (7.52, 7.61) | 1.82 (1.79, 1.86) | 0.77 (0.74, 0.80) |

Table S4. Global and regional expected lifetime risks (%) of osteoarthritis and its subtypes for women in 2021.

|  | OA | OA knee | OA hand | OA hip |
| --- | --- | --- | --- | --- |
| Global | 16.29 (16.28, 16.30) | 10.67 (10.66, 10.67) | 4.35 (4.34, 4.35) | 0.72 (0.71, 0.72) |
| Low SDI | 13.15 (13.11, 13.20) | 8.53 (8.50, 8.56) | 3.36 (3.33, 3.39) | 0.63 (0.61, 0.65) |
| Low-middle SDI | 14.58 (14.55, 14.60) | 9.33 (9.31, 9.35) | 4.05 (4.04, 4.07) | 0.59 (0.58, 0.59) |
| Middle SDI | 16.50 (16.48, 16.51) | 10.96 (10.94, 10.97) | 4.36 (4.35, 4.37) | 0.63 (0.62, 0.64) |
| High-middle SDI | 16.84 (16.83, 16.86) | 11.00 (10.98, 11.01) | 4.42 (4.41, 4.43) | 0.90 (0.89, 0.91) |
| High SDI | 18.31 (18.29, 18.32) | 11.84 (11.83, 11.85) | 5.03 (5.02, 5.04) | 0.98 (0.98, 0.98) |
| Central Europe, Eastern Europe, and Central Asia | 16.44 (16.41, 16.47) | 8.24 (8.22, 8.26) | 6.25 (6.23, 6.27) | 1.58 (1.56, 1.59) |
| Central Asia | 15.79 (15.70, 15.89) | 7.18 (7.12, 7.24) | 6.75 (6.70, 6.80) | 1.45 (1.39, 1.51) |
| Central Europe | 16.07 (16.02, 16.12) | 8.42 (8.39, 8.46) | 5.49 (5.46, 5.52) | 1.70 (1.67, 1.73) |
| Eastern Europe | 16.67 (16.63, 16.71) | 8.41 (8.39, 8.44) | 6.39 (6.37, 6.42) | 1.53 (1.51, 1.55) |
| High-income | 18.34 (18.33, 18.36) | 11.95 (11.94, 11.96) | 4.94 (4.93, 4.95) | 0.99 (0.99, 1.00) |
| Australasia | 18.15 (18.05, 18.25) | 11.98 (11.90, 12.06) | 4.64 (4.58, 4.71) | 0.98 (0.96, 1.00) |
| High-income Asia Pacific | 21.25 (21.21, 21.28) | 14.60 (14.57, 14.63) | 5.83 (5.80, 5.85) | 0.60 (0.60, 0.61) |
| High-income North America | 18.17 (18.14, 18.19) | 11.15 (11.13, 11.17) | 5.44 (5.42, 5.46) | 1.16 (1.16, 1.17) |
| Southern Latin America | 17.16 (17.09, 17.24) | 11.37 (11.32, 11.43) | 4.39 (4.34, 4.44) | 0.85 (0.84, 0.87) |
| Western Europe | 17.20 (17.18, 17.22) | 11.36 (11.34, 11.38) | 4.21 (4.20, 4.23) | 1.05 (1.04, 1.05) |
| Latin America and Caribbean | 16.95 (16.92, 16.99) | 10.97 (10.95, 10.99) | 4.75 (4.73, 4.77) | 0.74 (0.73, 0.75) |
| Andean Latin America | 16.94 (16.85, 17.03) | 11.28 (11.20, 11.35) | 4.49 (4.43, 4.54) | 0.70 (0.67, 0.73) |
| Caribbean | 16.06 (15.97, 16.16) | 10.69 (10.62, 10.77) | 4.09 (4.04, 4.15) | 0.72 (0.68, 0.75) |
| Central Latin America | 16.70 (16.65, 16.75) | 10.90 (10.86, 10.94) | 4.61 (4.57, 4.64) | 0.67 (0.66, 0.69) |
| Tropical Latin America | 17.38 (17.33, 17.43) | 11.03 (10.99, 11.06) | 5.07 (5.04, 5.11) | 0.82 (0.80, 0.84) |
| North Africa and Middle East | 14.52 (14.48, 14.57) | 9.34 (9.31, 9.37) | 3.80 (3.78, 3.83) | 0.70 (0.68, 0.72) |
| South Asia | 15.37 (15.35, 15.40) | 9.49 (9.47, 9.50) | 4.80 (4.78, 4.82) | 0.55 (0.54, 0.56) |
| Southeast Asia, East Asia, and Oceania | 16.56 (16.54, 16.58) | 11.78 (11.77, 11.80) | 3.61 (3.60, 3.62) | 0.55 (0.54, 0.56) |
| East Asia | 17.61 (17.59, 17.63) | 12.88 (12.87, 12.90) | 3.60 (3.58, 3.61) | 0.55 (0.55, 0.56) |
| Oceania | 14.02 (13.66, 14.37) | 9.92 (9.65, 10.19) | 2.91 (2.68, 3.13) | 0.54 (0.41, 0.67) |
| Southeast Asia | 13.50 (13.46, 13.53) | 8.64 (8.61, 8.66) | 3.59 (3.57, 3.62) | 0.54 (0.53, 0.55) |
| Sub-Saharan Africa | 13.55 (13.51, 13.59) | 8.45 (8.42, 8.48) | 3.76 (3.73, 3.79) | 0.75 (0.73, 0.77) |
| Central Sub-Saharan Africa | 12.99 (12.87, 13.11) | 8.02 (7.93, 8.10) | 3.68 (3.60, 3.76) | 0.70 (0.65, 0.76) |
| Eastern Sub-Saharan Africa | 13.05 (12.98, 13.11) | 8.10 (8.05, 8.15) | 3.59 (3.55, 3.63) | 0.75 (0.72, 0.78) |
| Southern Sub-Saharan Africa | 14.30 (14.20, 14.40) | 8.24 (8.17, 8.30) | 4.81 (4.74, 4.87) | 0.79 (0.74, 0.83) |
| Western Sub-Saharan Africa | 13.90 (13.83, 13.96) | 8.92 (8.87, 8.96) | 3.60 (3.55, 3.64) | 0.76 (0.73, 0.79) |

Table S5. National expected lifetime risk estimates (%) of osteoarthritis and its subtypes for men in 2021.

|  | OA | OA knee | OA hand | OA hip |
| --- | --- | --- | --- | --- |
| Afghanistan | 8.83 (8.62, 9.04) | 6.47 (6.31, 6.63) | 1.01 (0.92, 1.10) | 0.44 (0.35, 0.53) |
| Albania | 11.52 (11.02, 12.03) | 6.99 (6.68, 7.30) | 1.98 (1.76, 2.21) | 1.65 (1.28, 2.03) |
| Algeria | 11.60 (11.44, 11.76) | 7.97 (7.86, 8.09) | 1.92 (1.84, 2.00) | 0.69 (0.61, 0.78) |
| American Samoa | 12.68 (8.10, 17.26) | 7.89 (4.49, 11.29) | 3.02 (0.55, 5.49) | 0.86 (0.00, 2.97) |
| Andorra | 12.82 (10.97, 14.68) | 8.71 (7.17, 10.25) | 2.26 (1.35, 3.18) | 0.95 (0.51, 1.38) |
| Angola | 10.51 (10.24, 10.79) | 6.81 (6.62, 7.01) | 2.21 (2.07, 2.36) | 0.72 (0.57, 0.87) |
| Antigua and Barbuda | 13.17 (9.54, 16.79) | 8.89 (6.30, 11.47) | 2.61 (0.60, 4.63) | 0.80 (0.00, 2.59) |
| Argentina | 13.09 (12.99, 13.20) | 8.84 (8.76, 8.93) | 2.62 (2.57, 2.67) | 0.83 (0.80, 0.85) |
| Armenia | 11.00 (10.51, 11.49) | 6.27 (5.97, 6.57) | 2.10 (1.88, 2.32) | 1.72 (1.37, 2.07) |
| Australia | 14.12 (14.00, 14.24) | 9.63 (9.53, 9.72) | 2.68 (2.61, 2.74) | 0.96 (0.93, 0.99) |
| Austria | 12.56 (12.38, 12.74) | 8.56 (8.41, 8.71) | 2.19 (2.10, 2.28) | 0.91 (0.87, 0.95) |
| Azerbaijan | 10.65 (10.25, 11.05) | 6.14 (5.91, 6.38) | 2.08 (1.91, 2.26) | 1.53 (1.24, 1.83) |
| Bahamas | 12.80 (11.58, 14.02) | 8.71 (7.76, 9.65) | 2.48 (1.85, 3.11) | 0.79 (0.26, 1.31) |
| Bahrain | 11.94 (9.81, 14.06) | 8.18 (6.77, 9.59) | 2.03 (0.91, 3.15) | 0.71 (0.00, 1.99) |
| Bangladesh | 10.37 (10.29, 10.44) | 7.36 (7.30, 7.42) | 1.69 (1.64, 1.73) | 0.37 (0.34, 0.39) |
| Barbados | 13.67 (12.48, 14.86) | 9.25 (8.35, 10.16) | 2.65 (2.02, 3.27) | 0.89 (0.36, 1.43) |
| Belarus | 12.31 (12.08, 12.54) | 7.15 (7.00, 7.30) | 2.91 (2.80, 3.01) | 1.52 (1.36, 1.68) |
| Belgium | 12.58 (12.42, 12.74) | 8.54 (8.41, 8.68) | 2.25 (2.17, 2.33) | 0.89 (0.86, 0.93) |
| Belize | 13.32 (11.86, 14.77) | 9.16 (8.04, 10.27) | 2.37 (1.63, 3.12) | 0.89 (0.24, 1.53) |
| Benin | 11.03 (10.66, 11.40) | 7.62 (7.35, 7.89) | 1.76 (1.57, 1.95) | 0.78 (0.60, 0.97) |
| Bermuda | 14.03 (11.75, 16.31) | 9.52 (7.78, 11.27) | 2.67 (1.48, 3.87) | 0.95 (0.00, 1.96) |
| Bhutan | 10.97 (9.90, 12.04) | 7.95 (7.12, 8.78) | 1.56 (0.99, 2.13) | 0.44 (0.10, 0.78) |
| Bolivia (Plurinational State of) | 11.33 (11.11, 11.55) | 7.99 (7.82, 8.17) | 1.84 (1.74, 1.95) | 0.65 (0.56, 0.74) |
| Bosnia and Herzegovina | 12.07 (11.68, 12.46) | 6.98 (6.73, 7.22) | 2.53 (2.36, 2.71) | 1.72 (1.44, 2.01) |
| Botswana | 11.16 (10.52, 11.80) | 7.45 (6.99, 7.92) | 2.01 (1.69, 2.33) | 0.90 (0.55, 1.25) |
| Brazil | 13.80 (13.75, 13.86) | 9.03 (8.99, 9.07) | 3.02 (2.99, 3.05) | 0.91 (0.89, 0.94) |
| Brunei Darussalam | 13.94 (9.08, 18.80) | 9.78 (6.06, 13.50) | 2.78 (0.00, 5.72) | 0.65 (0.00, 1.58) |
| Bulgaria | 11.47 (11.25, 11.68) | 6.65 (6.51, 6.79) | 2.63 (2.53, 2.74) | 1.39 (1.24, 1.54) |
| Burkina Faso | 9.92 (9.65, 10.19) | 7.23 (7.04, 7.43) | 1.10 (0.97, 1.23) | 0.69 (0.56, 0.83) |
| Burundi | 9.63 (9.21, 10.05) | 6.74 (6.44, 7.03) | 1.32 (1.10, 1.53) | 0.72 (0.49, 0.94) |
| Cabo Verde | 11.64 (10.50, 12.78) | 7.98 (7.11, 8.85) | 1.89 (1.34, 2.44) | 0.87 (0.34, 1.40) |
| Cambodia | 8.69 (8.36, 9.01) | 5.46 (5.22, 5.69) | 1.82 (1.65, 2.00) | 0.49 (0.36, 0.62) |
| Cameroon | 10.83 (10.59, 11.07) | 7.62 (7.45, 7.79) | 1.56 (1.44, 1.68) | 0.78 (0.66, 0.91) |
| Canada | 10.97 (10.87, 11.06) | 6.18 (6.11, 6.26) | 3.09 (3.04, 3.14) | 0.74 (0.72, 0.76) |
| Central African Republic | 8.47 (7.87, 9.08) | 5.97 (5.54, 6.40) | 1.20 (0.89, 1.50) | 0.56 (0.24, 0.88) |
| Chad | 9.76 (9.41, 10.11) | 6.99 (6.74, 7.24) | 1.25 (1.07, 1.42) | 0.65 (0.47, 0.83) |
| Chile | 13.48 (13.33, 13.63) | 9.21 (9.09, 9.34) | 2.54 (2.45, 2.62) | 0.89 (0.86, 0.93) |
| China | 11.99 (11.97, 12.02) | 7.94 (7.93, 7.96) | 2.54 (2.52, 2.55) | 0.58 (0.58, 0.59) |
| Colombia | 13.06 (12.97, 13.16) | 8.92 (8.85, 9.00) | 2.51 (2.46, 2.56) | 0.73 (0.70, 0.77) |
| Comoros | 10.79 (9.53, 12.05) | 7.37 (6.48, 8.27) | 1.66 (1.03, 2.29) | 0.88 (0.21, 1.54) |
| Congo | 10.86 (10.25, 11.48) | 7.20 (6.78, 7.63) | 2.03 (1.71, 2.35) | 0.82 (0.48, 1.16) |
| Cook Islands | 12.99 (7.42, 18.57) | 8.08 (3.89, 12.26) | 3.08 (0.07, 6.10) | 0.90 (0.00, 3.37) |
| Costa Rica | 13.35 (13.03, 13.68) | 9.15 (8.89, 9.41) | 2.50 (2.34, 2.67) | 0.79 (0.66, 0.91) |
| Croatia | 12.60 (12.22, 12.99) | 7.21 (6.98, 7.43) | 2.74 (2.56, 2.91) | 1.82 (1.53, 2.11) |
| Cuba | 12.76 (12.61, 12.90) | 8.82 (8.70, 8.94) | 2.27 (2.20, 2.35) | 0.79 (0.74, 0.85) |
| Cyprus | 12.31 (10.00, 14.62) | 8.37 (6.60, 10.14) | 2.19 (0.83, 3.55) | 0.86 (0.44, 1.28) |
| Czechia | 12.92 (12.72, 13.12) | 7.28 (7.15, 7.40) | 2.94 (2.84, 3.03) | 1.89 (1.75, 2.03) |
| CÃ´te d'Ivoire | 10.80 (10.51, 11.10) | 7.51 (7.31, 7.72) | 1.66 (1.51, 1.82) | 0.76 (0.61, 0.92) |
| Democratic People's Republic of Korea | 10.65 (10.37, 10.94) | 7.25 (7.04, 7.46) | 2.06 (1.90, 2.22) | 0.46 (0.34, 0.57) |
| Democratic Republic of the Congo | 9.73 (9.58, 9.89) | 6.73 (6.62, 6.84) | 1.48 (1.40, 1.56) | 0.70 (0.62, 0.78) |
| Denmark | 12.70 (12.47, 12.93) | 8.66 (8.47, 8.85) | 2.21 (2.09, 2.32) | 0.97 (0.91, 1.02) |
| Djibouti | 10.77 (9.34, 12.20) | 7.20 (6.22, 8.18) | 1.89 (1.15, 2.64) | 0.82 (0.01, 1.63) |
| Dominica | 12.57 (9.61, 15.54) | 8.71 (6.45, 10.97) | 2.24 (0.71, 3.78) | 0.77 (0.00, 2.08) |
| Dominican Republic | 13.17 (12.92, 13.43) | 8.96 (8.77, 9.16) | 2.49 (2.35, 2.62) | 0.84 (0.72, 0.95) |
| Ecuador | 12.89 (12.67, 13.11) | 8.96 (8.79, 9.12) | 2.15 (2.04, 2.27) | 0.86 (0.76, 0.96) |
| Egypt | 10.96 (10.88, 11.05) | 7.72 (7.65, 7.79) | 1.60 (1.56, 1.64) | 0.66 (0.62, 0.70) |
| El Salvador | 12.67 (12.40, 12.95) | 8.73 (8.51, 8.96) | 2.34 (2.20, 2.47) | 0.72 (0.62, 0.82) |
| Equatorial Guinea | 11.02 (9.78, 12.25) | 7.37 (6.49, 8.25) | 1.97 (1.34, 2.59) | 0.88 (0.20, 1.55) |
| Eritrea | 9.41 (8.57, 10.25) | 6.55 (5.98, 7.11) | 1.36 (0.92, 1.80) | 0.67 (0.19, 1.14) |
| Estonia | 13.64 (12.96, 14.32) | 7.74 (7.32, 8.16) | 3.24 (2.93, 3.55) | 1.91 (1.40, 2.42) |
| Eswatini | 9.77 (8.93, 10.62) | 6.70 (6.07, 7.33) | 1.59 (1.17, 2.00) | 0.75 (0.30, 1.21) |
| Ethiopia | 11.52 (11.39, 11.64) | 7.17 (7.08, 7.26) | 2.69 (2.62, 2.76) | 0.87 (0.81, 0.94) |
| Fiji | 11.16 (9.92, 12.39) | 7.01 (6.09, 7.94) | 2.66 (2.00, 3.33) | 0.64 (0.09, 1.19) |
| Finland | 12.64 (12.41, 12.86) | 8.61 (8.43, 8.80) | 2.20 (2.09, 2.31) | 0.92 (0.87, 0.97) |
| France | 12.57 (12.50, 12.63) | 8.51 (8.45, 8.56) | 2.23 (2.20, 2.26) | 0.94 (0.92, 0.96) |
| Gabon | 11.12 (10.25, 11.99) | 7.51 (6.89, 8.12) | 1.88 (1.44, 2.33) | 0.90 (0.42, 1.38) |
| Gambia | 10.73 (9.90, 11.56) | 7.34 (6.74, 7.94) | 1.84 (1.41, 2.27) | 0.72 (0.29, 1.14) |
| Georgia | 10.21 (9.87, 10.55) | 6.02 (5.80, 6.24) | 1.80 (1.65, 1.95) | 1.49 (1.26, 1.71) |
| Germany | 12.65 (12.58, 12.71) | 8.60 (8.55, 8.65) | 2.23 (2.20, 2.26) | 0.93 (0.91, 0.94) |
| Ghana | 11.14 (10.91, 11.38) | 7.59 (7.42, 7.75) | 1.95 (1.83, 2.07) | 0.77 (0.65, 0.90) |
| Greece | 12.28 (12.14, 12.42) | 8.51 (8.39, 8.62) | 2.04 (1.98, 2.11) | 0.82 (0.79, 0.86) |
| Greenland | 10.83 (5.95, 15.72) | 6.32 (2.58, 10.05) | 2.92 (0.08, 5.76) | 0.74 (0.00, 1.60) |
| Grenada | 12.12 (2.63, 21.61) | 8.37 (1.87, 14.88) | 2.25 (0.00, 7.72) | 0.68 (0.00, 5.60) |
| Guam | 13.00 (11.37, 14.63) | 8.00 (6.72, 9.28) | 3.17 (2.32, 4.02) | 0.90 (0.26, 1.53) |
| Guatemala | 11.83 (11.49, 12.18) | 8.31 (8.06, 8.55) | 2.03 (1.84, 2.23) | 0.62 (0.45, 0.78) |
| Guinea | 10.01 (9.72, 10.29) | 7.15 (6.94, 7.36) | 1.30 (1.16, 1.44) | 0.68 (0.55, 0.81) |
| Guinea-Bissau | 9.40 (8.35, 10.46) | 6.70 (5.95, 7.46) | 1.31 (0.77, 1.84) | 0.59 (0.06, 1.12) |
| Guyana | 11.68 (10.85, 12.52) | 8.05 (7.39, 8.71) | 2.17 (1.75, 2.58) | 0.67 (0.34, 1.00) |
| Haiti | 10.44 (10.17, 10.72) | 7.45 (7.24, 7.66) | 1.63 (1.49, 1.77) | 0.54 (0.43, 0.65) |
| Honduras | 11.71 (11.40, 12.03) | 8.24 (8.00, 8.48) | 2.01 (1.85, 2.17) | 0.60 (0.47, 0.73) |
| Hungary | 12.62 (12.41, 12.84) | 7.14 (7.01, 7.28) | 2.85 (2.76, 2.95) | 1.82 (1.67, 1.98) |
| Iceland | 13.12 (12.11, 14.14) | 8.87 (8.04, 9.71) | 2.30 (1.79, 2.80) | 1.05 (0.80, 1.29) |
| India | 10.65 (10.62, 10.67) | 7.49 (7.46, 7.51) | 1.81 (1.79, 1.82) | 0.40 (0.39, 0.41) |
| Indonesia | 10.26 (10.19, 10.34) | 5.91 (5.86, 5.96) | 2.81 (2.77, 2.86) | 0.63 (0.60, 0.66) |
| Iran (Islamic Republic of) | 11.90 (11.81, 11.99) | 7.94 (7.87, 8.00) | 2.17 (2.12, 2.22) | 0.81 (0.76, 0.85) |
| Iraq | 10.98 (10.80, 11.15) | 7.66 (7.53, 7.79) | 1.69 (1.61, 1.77) | 0.64 (0.55, 0.73) |
| Ireland | 12.76 (12.45, 13.06) | 8.70 (8.46, 8.95) | 2.22 (2.06, 2.37) | 0.93 (0.87, 1.00) |
| Israel | 12.71 (12.49, 12.93) | 8.65 (8.47, 8.83) | 2.28 (2.17, 2.38) | 0.87 (0.82, 0.92) |
| Italy | 13.10 (13.03, 13.17) | 8.62 (8.57, 8.68) | 2.71 (2.67, 2.75) | 0.88 (0.86, 0.89) |
| Jamaica | 13.01 (12.63, 13.39) | 8.96 (8.65, 9.26) | 2.34 (2.15, 2.52) | 0.83 (0.68, 0.97) |
| Japan | 14.67 (14.62, 14.71) | 9.84 (9.81, 9.88) | 3.41 (3.39, 3.44) | 0.66 (0.65, 0.67) |
| Jordan | 12.29 (11.93, 12.65) | 8.34 (8.08, 8.60) | 2.09 (1.91, 2.26) | 0.83 (0.64, 1.01) |
| Kazakhstan | 10.60 (10.34, 10.87) | 6.05 (5.89, 6.22) | 2.23 (2.11, 2.35) | 1.45 (1.26, 1.65) |
| Kenya | 11.50 (11.29, 11.71) | 7.39 (7.25, 7.54) | 2.37 (2.25, 2.49) | 0.93 (0.82, 1.04) |
| Kiribati | 10.92 (5.69, 16.16) | 6.84 (3.01, 10.66) | 2.62 (0.00, 5.47) | 0.63 (0.00, 3.01) |
| Kuwait | 13.03 (12.52, 13.55) | 8.82 (8.44, 9.21) | 2.20 (1.95, 2.45) | 0.95 (0.68, 1.21) |
| Kyrgyzstan | 10.51 (9.78, 11.24) | 5.98 (5.57, 6.40) | 2.11 (1.79, 2.44) | 1.52 (0.97, 2.08) |
| Lao People's Democratic Republic | 9.05 (8.60, 9.49) | 5.65 (5.32, 5.98) | 1.92 (1.69, 2.16) | 0.53 (0.35, 0.71) |
| Latvia | 13.00 (12.49, 13.52) | 7.45 (7.13, 7.78) | 3.04 (2.80, 3.28) | 1.77 (1.39, 2.14) |
| Lebanon | 11.85 (11.58, 12.13) | 8.11 (7.90, 8.33) | 1.99 (1.87, 2.12) | 0.72 (0.60, 0.84) |
| Lesotho | 8.88 (8.37, 9.39) | 6.08 (5.69, 6.48) | 1.50 (1.26, 1.75) | 0.62 (0.38, 0.86) |
| Liberia | 11.11 (10.56, 11.66) | 7.71 (7.31, 8.12) | 1.70 (1.43, 1.98) | 0.81 (0.54, 1.09) |
| Libya | 11.62 (11.29, 11.95) | 7.95 (7.70, 8.21) | 1.94 (1.79, 2.10) | 0.73 (0.57, 0.89) |
| Lithuania | 12.99 (12.56, 13.42) | 7.45 (7.18, 7.72) | 3.03 (2.84, 3.23) | 1.76 (1.44, 2.07) |
| Luxembourg | 12.72 (11.88, 13.57) | 8.63 (7.94, 9.32) | 2.26 (1.82, 2.70) | 0.92 (0.73, 1.11) |
| Madagascar | 9.55 (9.27, 9.83) | 6.80 (6.60, 7.00) | 1.16 (1.02, 1.30) | 0.72 (0.57, 0.86) |
| Malawi | 9.28 (9.02, 9.54) | 6.57 (6.38, 6.77) | 1.24 (1.12, 1.37) | 0.67 (0.54, 0.80) |
| Malaysia | 10.69 (10.54, 10.84) | 6.24 (6.13, 6.35) | 2.83 (2.75, 2.91) | 0.67 (0.61, 0.73) |
| Maldives | 11.17 (9.59, 12.75) | 6.42 (5.25, 7.59) | 3.09 (2.23, 3.95) | 0.68 (0.04, 1.33) |
| Mali | 10.05 (9.75, 10.34) | 7.11 (6.90, 7.32) | 1.42 (1.27, 1.57) | 0.66 (0.51, 0.82) |
| Malta | 12.81 (11.96, 13.66) | 8.68 (7.98, 9.37) | 2.29 (1.85, 2.73) | 0.92 (0.73, 1.12) |
| Marshall Islands | 11.21 (3.86, 18.57) | 6.82 (1.42, 12.22) | 2.95 (0.00, 6.98) | 0.62 (0.00, 3.92) |
| Mauritania | 11.83 (11.27, 12.39) | 8.17 (7.77, 8.58) | 1.81 (1.53, 2.09) | 0.92 (0.63, 1.21) |
| Mauritius | 10.83 (10.23, 11.44) | 6.25 (5.80, 6.71) | 2.98 (2.65, 3.30) | 0.67 (0.44, 0.91) |
| Mexico | 14.11 (14.04, 14.18) | 9.14 (9.08, 9.19) | 3.33 (3.30, 3.37) | 0.84 (0.81, 0.87) |
| Micronesia (Federated States of) | 11.42 (7.28, 15.56) | 7.11 (4.03, 10.19) | 2.77 (0.54, 4.99) | 0.69 (0.00, 2.52) |
| Monaco | 12.69 (10.41, 14.97) | 8.65 (6.75, 10.55) | 2.19 (1.08, 3.30) | 0.98 (0.38, 1.57) |
| Mongolia | 10.40 (8.83, 11.96) | 5.83 (4.99, 6.67) | 2.28 (1.57, 2.98) | 1.45 (0.19, 2.70) |
| Montenegro | 11.57 (10.86, 12.28) | 6.79 (6.29, 7.29) | 2.63 (2.30, 2.96) | 1.34 (0.90, 1.78) |
| Morocco | 11.16 (11.01, 11.31) | 7.76 (7.65, 7.86) | 1.72 (1.65, 1.80) | 0.68 (0.60, 0.75) |
| Mozambique | 8.95 (8.73, 9.17) | 6.34 (6.18, 6.51) | 1.18 (1.08, 1.29) | 0.63 (0.52, 0.74) |
| Myanmar | 9.20 (9.08, 9.32) | 5.56 (5.47, 5.65) | 2.23 (2.16, 2.29) | 0.52 (0.47, 0.56) |
| Namibia | 10.29 (9.69, 10.88) | 7.03 (6.59, 7.48) | 1.68 (1.39, 1.97) | 0.78 (0.47, 1.09) |
| Nauru | 11.04 (0.00, 24.73) | 6.86 (0.00, 17.04) | 2.71 (0.00, 10.10) | 0.65 (0.00, 6.76) |
| Nepal | 9.74 (9.59, 9.90) | 7.22 (7.09, 7.34) | 1.22 (1.14, 1.30) | 0.36 (0.31, 0.40) |
| Netherlands | 13.01 (12.87, 13.14) | 8.99 (8.88, 9.10) | 2.23 (2.16, 2.30) | 0.92 (0.88, 0.95) |
| New Zealand | 14.58 (14.28, 14.88) | 9.57 (9.33, 9.81) | 3.25 (3.07, 3.43) | 0.95 (0.88, 1.02) |
| Nicaragua | 12.38 (11.91, 12.85) | 8.66 (8.31, 9.00) | 2.13 (1.88, 2.39) | 0.68 (0.47, 0.90) |
| Niger | 9.98 (9.65, 10.30) | 7.17 (6.94, 7.40) | 1.23 (1.07, 1.40) | 0.68 (0.52, 0.85) |
| Nigeria | 11.58 (11.49, 11.68) | 7.76 (7.70, 7.83) | 2.14 (2.09, 2.19) | 0.80 (0.76, 0.85) |
| Niue | 12.27 (0.00, 30.82) | 7.61 (0.00, 21.61) | 2.97 (0.00, 12.93) | 0.79 (0.00, 8.85) |
| North Macedonia | 11.37 (9.94, 12.81) | 6.73 (5.99, 7.48) | 2.43 (1.79, 3.08) | 1.39 (0.19, 2.59) |
| Northern Mariana Islands | 12.25 (7.38, 17.13) | 7.62 (3.98, 11.25) | 2.96 (0.36, 5.57) | 0.79 (0.00, 2.98) |
| Norway | 13.36 (13.10, 13.61) | 8.68 (8.47, 8.89) | 2.87 (2.73, 3.02) | 0.92 (0.86, 0.98) |
| Oman | 11.62 (10.80, 12.44) | 7.96 (7.38, 8.55) | 1.99 (1.58, 2.40) | 0.68 (0.23, 1.12) |
| Pakistan | 10.07 (10.00, 10.14) | 7.00 (6.95, 7.06) | 1.67 (1.63, 1.71) | 0.42 (0.40, 0.45) |
| Palau | 12.47 (3.92, 21.02) | 7.72 (1.50, 13.95) | 3.02 (0.00, 7.70) | 0.83 (0.00, 4.86) |
| Palestine | 11.17 (10.16, 12.19) | 7.69 (7.01, 8.38) | 1.87 (1.34, 2.40) | 0.63 (0.04, 1.21) |
| Panama | 13.25 (12.89, 13.61) | 9.05 (8.76, 9.33) | 2.52 (2.34, 2.70) | 0.76 (0.62, 0.90) |
| Papua New Guinea | 9.73 (9.35, 10.11) | 6.45 (6.16, 6.74) | 1.86 (1.66, 2.06) | 0.54 (0.39, 0.69) |
| Paraguay | 12.40 (12.10, 12.69) | 8.55 (8.32, 8.78) | 2.24 (2.09, 2.39) | 0.74 (0.62, 0.85) |
| Peru | 12.31 (12.20, 12.41) | 8.49 (8.40, 8.58) | 2.18 (2.13, 2.23) | 0.76 (0.72, 0.80) |
| Philippines | 9.98 (9.90, 10.07) | 5.35 (5.29, 5.41) | 3.13 (3.08, 3.18) | 0.60 (0.57, 0.64) |
| Poland | 13.44 (13.32, 13.57) | 7.33 (7.25, 7.41) | 3.31 (3.25, 3.37) | 2.02 (1.93, 2.11) |
| Portugal | 12.30 (12.14, 12.46) | 8.39 (8.26, 8.52) | 2.13 (2.05, 2.21) | 0.87 (0.84, 0.91) |
| Puerto Rico | 14.50 (14.21, 14.78) | 9.83 (9.60, 10.05) | 2.70 (2.56, 2.84) | 1.05 (0.94, 1.16) |
| Qatar | 12.88 (10.30, 15.45) | 8.78 (7.09, 10.47) | 2.15 (0.81, 3.48) | 0.89 (0.00, 2.46) |
| Republic of Korea | 14.52 (14.41, 14.63) | 10.39 (10.30, 10.48) | 2.74 (2.68, 2.81) | 0.63 (0.61, 0.65) |
| Republic of Moldova | 12.49 (12.09, 12.88) | 7.38 (7.13, 7.63) | 2.54 (2.37, 2.72) | 1.78 (1.49, 2.06) |
| Romania | 11.81 (11.67, 11.95) | 6.94 (6.85, 7.03) | 2.34 (2.28, 2.40) | 1.69 (1.59, 1.78) |
| Russian Federation | 13.69 (13.63, 13.76) | 7.53 (7.49, 7.57) | 3.57 (3.54, 3.60) | 1.92 (1.87, 1.96) |
| Rwanda | 9.90 (9.51, 10.29) | 7.05 (6.77, 7.32) | 1.19 (1.00, 1.39) | 0.78 (0.57, 0.99) |
| Saint Kitts and Nevis | 12.99 (9.40, 16.58) | 8.88 (6.16, 11.60) | 2.45 (0.58, 4.32) | 0.80 (0.00, 2.42) |
| Saint Lucia | 12.83 (11.25, 14.40) | 8.84 (7.63, 10.06) | 2.33 (1.52, 3.15) | 0.78 (0.10, 1.47) |
| Saint Vincent and the Grenadines | 12.72 (10.07, 15.37) | 8.78 (6.87, 10.68) | 2.30 (0.85, 3.75) | 0.78 (0.00, 2.06) |
| Samoa | 12.04 (9.52, 14.56) | 7.53 (5.65, 9.41) | 2.84 (1.48, 4.20) | 0.76 (0.00, 1.88) |
| San Marino | 13.07 (10.51, 15.63) | 8.90 (6.75, 11.05) | 2.29 (1.07, 3.51) | 0.96 (0.30, 1.62) |
| Sao Tome and Principe | 11.93 (9.01, 14.84) | 7.93 (5.87, 9.99) | 2.29 (0.77, 3.81) | 0.85 (0.00, 2.40) |
| Saudi Arabia | 12.32 (12.03, 12.61) | 8.37 (8.17, 8.57) | 2.10 (1.95, 2.24) | 0.83 (0.67, 0.99) |
| Senegal | 10.77 (10.49, 11.04) | 7.49 (7.29, 7.69) | 1.66 (1.52, 1.80) | 0.74 (0.61, 0.88) |
| Serbia | 11.98 (11.79, 12.16) | 7.03 (6.90, 7.16) | 2.46 (2.37, 2.55) | 1.64 (1.52, 1.76) |
| Seychelles | 10.87 (8.15, 13.58) | 6.28 (4.26, 8.29) | 2.99 (1.52, 4.46) | 0.67 (0.00, 1.79) |
| Sierra Leone | 10.60 (10.15, 11.05) | 7.37 (7.05, 7.69) | 1.61 (1.38, 1.84) | 0.74 (0.51, 0.97) |
| Singapore | 14.65 (14.33, 14.98) | 10.26 (9.99, 10.52) | 2.94 (2.76, 3.12) | 0.67 (0.61, 0.74) |
| Slovakia | 12.50 (12.20, 12.81) | 7.08 (6.88, 7.28) | 2.86 (2.72, 3.00) | 1.75 (1.53, 1.97) |
| Slovenia | 13.23 (12.70, 13.75) | 7.43 (7.11, 7.75) | 2.93 (2.70, 3.17) | 2.03 (1.63, 2.42) |
| Solomon Islands | 10.64 (8.74, 12.54) | 6.76 (5.37, 8.14) | 2.41 (1.37, 3.45) | 0.61 (0.00, 1.45) |
| Somalia | 8.34 (7.97, 8.70) | 5.99 (5.73, 6.25) | 1.03 (0.85, 1.22) | 0.55 (0.35, 0.74) |
| South Africa | 13.01 (12.88, 13.14) | 8.38 (8.29, 8.48) | 2.61 (2.54, 2.68) | 1.22 (1.15, 1.30) |
| South Sudan | 9.45 (9.12, 9.78) | 6.77 (6.52, 7.02) | 1.12 (0.97, 1.27) | 0.73 (0.57, 0.89) |
| Spain | 12.25 (12.18, 12.33) | 8.18 (8.12, 8.25) | 2.20 (2.16, 2.24) | 0.95 (0.93, 0.97) |
| Sri Lanka | 10.17 (9.96, 10.38) | 6.03 (5.88, 6.19) | 2.56 (2.44, 2.67) | 0.61 (0.52, 0.69) |
| Sudan | 10.58 (10.42, 10.75) | 7.45 (7.33, 7.58) | 1.52 (1.44, 1.59) | 0.62 (0.54, 0.69) |
| Suriname | 12.95 (12.00, 13.91) | 8.78 (8.03, 9.52) | 2.52 (2.03, 3.00) | 0.81 (0.41, 1.21) |
| Sweden | 12.25 (12.08, 12.42) | 7.76 (7.63, 7.90) | 2.55 (2.46, 2.65) | 0.99 (0.95, 1.03) |
| Switzerland | 12.58 (12.40, 12.76) | 8.54 (8.39, 8.69) | 2.26 (2.17, 2.35) | 0.87 (0.82, 0.91) |
| Syrian Arab Republic | 11.21 (10.96, 11.46) | 7.75 (7.57, 7.93) | 1.81 (1.68, 1.93) | 0.67 (0.54, 0.80) |
| Taiwan (Province of China) | 12.88 (12.77, 13.00) | 8.30 (8.20, 8.39) | 3.05 (2.99, 3.11) | 0.62 (0.58, 0.66) |
| Tajikistan | 9.85 (9.33, 10.37) | 5.71 (5.41, 6.02) | 1.82 (1.58, 2.05) | 1.41 (1.03, 1.79) |
| Thailand | 10.72 (10.65, 10.80) | 6.68 (6.62, 6.74) | 2.35 (2.32, 2.39) | 0.69 (0.66, 0.72) |
| Timor-Leste | 9.10 (8.12, 10.07) | 5.47 (4.75, 6.19) | 2.22 (1.70, 2.75) | 0.50 (0.11, 0.88) |
| Togo | 10.37 (9.80, 10.94) | 7.22 (6.82, 7.62) | 1.61 (1.31, 1.90) | 0.70 (0.40, 1.00) |
| Tokelau | 12.29 (0.00, 31.13) | 7.63 (0.00, 22.14) | 2.96 (0.00, 12.95) | 0.78 (0.00, 8.32) |
| Tonga | 11.92 (8.79, 15.06) | 7.60 (5.26, 9.94) | 2.61 (0.93, 4.29) | 0.78 (0.00, 2.15) |
| Trinidad and Tobago | 12.98 (12.46, 13.51) | 8.87 (8.46, 9.28) | 2.45 (2.18, 2.72) | 0.82 (0.60, 1.04) |
| Tunisia | 11.31 (11.10, 11.52) | 7.76 (7.60, 7.92) | 1.91 (1.81, 2.01) | 0.66 (0.56, 0.76) |
| Turkmenistan | 10.41 (9.87, 10.95) | 6.00 (5.67, 6.33) | 2.04 (1.80, 2.28) | 1.50 (1.11, 1.89) |
| Tuvalu | 11.55 (1.94, 21.15) | 7.16 (0.00, 14.32) | 2.82 (0.00, 7.99) | 0.69 (0.00, 4.86) |
| TÃ¼rkiye | 11.69 (11.61, 11.78) | 8.13 (8.06, 8.19) | 1.78 (1.74, 1.82) | 0.75 (0.71, 0.79) |
| Uganda | 9.65 (9.43, 9.87) | 6.81 (6.65, 6.97) | 1.27 (1.16, 1.38) | 0.73 (0.61, 0.84) |
| Ukraine | 13.23 (13.11, 13.34) | 7.44 (7.36, 7.51) | 3.28 (3.22, 3.33) | 1.80 (1.71, 1.88) |
| United Arab Emirates | 12.78 (11.25, 14.31) | 8.68 (7.67, 9.69) | 2.16 (1.37, 2.95) | 0.90 (0.00, 1.82) |
| United Kingdom | 13.79 (13.72, 13.86) | 9.28 (9.22, 9.34) | 2.66 (2.62, 2.70) | 1.00 (0.99, 1.02) |
| United Republic of Tanzania | 10.42 (10.26, 10.58) | 7.21 (7.10, 7.33) | 1.50 (1.42, 1.58) | 0.83 (0.75, 0.92) |
| United States Virgin Islands | 13.90 (11.19, 16.60) | 9.48 (7.54, 11.42) | 2.57 (1.09, 4.05) | 0.96 (0.00, 2.30) |
| United States of America | 14.65 (14.62, 14.68) | 9.27 (9.24, 9.29) | 3.67 (3.65, 3.69) | 1.04 (1.04, 1.05) |
| Uruguay | 13.03 (12.73, 13.33) | 8.87 (8.62, 9.12) | 2.50 (2.35, 2.65) | 0.85 (0.78, 0.92) |
| Uzbekistan | 10.93 (10.60, 11.26) | 6.22 (6.03, 6.40) | 2.15 (2.01, 2.30) | 1.65 (1.40, 1.91) |
| Vanuatu | 9.95 (7.42, 12.48) | 6.71 (4.85, 8.57) | 1.74 (0.40, 3.09) | 0.60 (0.00, 1.71) |
| Venezuela (Bolivarian Republic of) | 12.54 (12.41, 12.67) | 8.61 (8.51, 8.71) | 2.39 (2.33, 2.46) | 0.68 (0.63, 0.72) |
| Viet Nam | 9.16 (9.05, 9.27) | 5.62 (5.54, 5.70) | 2.07 (2.01, 2.13) | 0.52 (0.48, 0.57) |
| Yemen | 9.63 (9.41, 9.86) | 6.79 (6.62, 6.95) | 1.41 (1.30, 1.52) | 0.49 (0.39, 0.60) |
| Zambia | 9.38 (9.14, 9.61) | 6.54 (6.37, 6.72) | 1.39 (1.27, 1.50) | 0.66 (0.55, 0.78) |
| Zimbabwe | 9.09 (8.86, 9.31) | 6.51 (6.34, 6.68) | 1.13 (1.03, 1.23) | 0.67 (0.56, 0.77) |

Table S6. National expected lifetime risk estimates (%) of osteoarthritis and its subtypes for women in 2021.

|  | OA | OA knee | OA hand | OA hip |
| --- | --- | --- | --- | --- |
| Afghanistan | 11.16 (10.90, 11.42) | 7.80 (7.62, 7.99) | 2.20 (2.04, 2.35) | 0.44 (0.33, 0.56) |
| Albania | 14.28 (13.87, 14.69) | 8.18 (7.91, 8.45) | 3.97 (3.74, 4.21) | 1.51 (1.25, 1.76) |
| Algeria | 14.42 (14.17, 14.67) | 9.29 (9.13, 9.46) | 3.83 (3.66, 3.99) | 0.62 (0.48, 0.75) |
| American Samoa | 16.69 (12.21, 21.17) | 11.37 (8.08, 14.67) | 4.01 (1.06, 6.96) | 0.74 (0.00, 2.55) |
| Andorra | 17.13 (15.31, 18.94) | 11.42 (9.94, 12.90) | 4.02 (2.93, 5.11) | 1.08 (0.66, 1.49) |
| Angola | 14.02 (13.74, 14.31) | 8.10 (7.91, 8.30) | 4.70 (4.52, 4.89) | 0.73 (0.58, 0.87) |
| Antigua and Barbuda | 16.63 (14.07, 19.20) | 10.80 (8.92, 12.68) | 4.62 (2.94, 6.31) | 0.70 (0.00, 1.72) |
| Argentina | 17.08 (16.99, 17.17) | 11.27 (11.19, 11.34) | 4.44 (4.38, 4.50) | 0.83 (0.82, 0.85) |
| Armenia | 15.89 (15.46, 16.31) | 7.34 (7.07, 7.61) | 6.59 (6.34, 6.85) | 1.52 (1.24, 1.80) |
| Australia | 18.08 (17.97, 18.19) | 12.00 (11.92, 12.09) | 4.53 (4.46, 4.60) | 0.99 (0.97, 1.01) |
| Austria | 16.84 (16.68, 17.00) | 11.26 (11.13, 11.39) | 3.93 (3.83, 4.02) | 1.04 (1.00, 1.07) |
| Azerbaijan | 15.78 (15.51, 16.05) | 7.25 (7.07, 7.42) | 6.68 (6.53, 6.84) | 1.44 (1.27, 1.61) |
| Bahamas | 16.61 (15.45, 17.77) | 10.87 (9.99, 11.76) | 4.52 (3.78, 5.25) | 0.72 (0.29, 1.15) |
| Bahrain | 15.20 (13.18, 17.21) | 9.67 (8.40, 10.95) | 4.13 (2.81, 5.45) | 0.73 (0.00, 1.88) |
| Bangladesh | 13.99 (13.90, 14.09) | 8.97 (8.91, 9.04) | 3.87 (3.81, 3.93) | 0.50 (0.47, 0.53) |
| Barbados | 17.04 (15.97, 18.10) | 11.13 (10.33, 11.94) | 4.63 (3.95, 5.32) | 0.76 (0.36, 1.17) |
| Belarus | 16.09 (15.92, 16.26) | 8.52 (8.40, 8.64) | 5.68 (5.58, 5.79) | 1.47 (1.37, 1.56) |
| Belgium | 16.83 (16.69, 16.98) | 11.19 (11.07, 11.31) | 4.02 (3.93, 4.11) | 1.01 (0.98, 1.05) |
| Belize | 16.69 (15.17, 18.21) | 11.13 (9.98, 12.27) | 4.24 (3.28, 5.20) | 0.77 (0.19, 1.36) |
| Benin | 13.98 (13.62, 14.34) | 9.06 (8.81, 9.32) | 3.48 (3.25, 3.71) | 0.79 (0.62, 0.96) |
| Bermuda | 17.80 (15.83, 19.77) | 11.64 (10.09, 13.18) | 4.79 (3.56, 6.03) | 0.86 (0.16, 1.56) |
| Bhutan | 14.34 (13.21, 15.47) | 9.51 (8.68, 10.33) | 3.55 (2.80, 4.30) | 0.59 (0.22, 0.96) |
| Bolivia (Plurinational State of) | 15.78 (15.54, 16.02) | 10.72 (10.53, 10.90) | 3.97 (3.82, 4.13) | 0.60 (0.51, 0.68) |
| Bosnia and Herzegovina | 15.19 (14.85, 15.54) | 8.21 (7.99, 8.43) | 4.91 (4.70, 5.11) | 1.56 (1.34, 1.77) |
| Botswana | 13.79 (13.22, 14.35) | 8.38 (7.97, 8.79) | 4.09 (3.74, 4.44) | 0.77 (0.52, 1.03) |
| Brazil | 17.40 (17.35, 17.45) | 11.03 (11.00, 11.07) | 5.09 (5.06, 5.13) | 0.82 (0.80, 0.84) |
| Brunei Darussalam | 20.39 (17.15, 23.63) | 14.43 (12.34, 16.51) | 5.07 (2.34, 7.81) | 0.63 (0.30, 0.96) |
| Bulgaria | 15.19 (14.98, 15.39) | 8.09 (7.96, 8.23) | 5.22 (5.09, 5.34) | 1.40 (1.27, 1.53) |
| Burkina Faso | 12.41 (12.13, 12.69) | 8.66 (8.47, 8.86) | 2.31 (2.13, 2.48) | 0.71 (0.58, 0.83) |
| Burundi | 11.94 (11.50, 12.39) | 7.83 (7.53, 8.13) | 2.75 (2.47, 3.03) | 0.68 (0.47, 0.89) |
| Cabo Verde | 14.86 (13.79, 15.93) | 9.52 (8.73, 10.31) | 3.78 (3.12, 4.43) | 0.90 (0.45, 1.35) |
| Cambodia | 12.04 (11.76, 12.32) | 8.18 (7.98, 8.38) | 2.66 (2.48, 2.84) | 0.45 (0.35, 0.55) |
| Cameroon | 13.69 (13.44, 13.95) | 9.11 (8.94, 9.29) | 3.13 (2.97, 3.29) | 0.80 (0.67, 0.92) |
| Canada | 13.91 (13.83, 13.99) | 7.75 (7.69, 7.81) | 4.52 (4.47, 4.58) | 0.83 (0.81, 0.84) |
| Central African Republic | 11.39 (10.74, 12.05) | 7.39 (6.95, 7.83) | 2.81 (2.39, 3.24) | 0.58 (0.26, 0.90) |
| Chad | 12.08 (11.67, 12.48) | 8.24 (7.96, 8.52) | 2.50 (2.25, 2.76) | 0.64 (0.45, 0.82) |
| Chile | 17.37 (17.22, 17.52) | 11.61 (11.50, 11.73) | 4.30 (4.19, 4.40) | 0.89 (0.87, 0.92) |
| China | 17.65 (17.62, 17.67) | 12.90 (12.89, 12.92) | 3.61 (3.60, 3.63) | 0.55 (0.55, 0.56) |
| Colombia | 16.48 (16.38, 16.57) | 10.93 (10.85, 11.00) | 4.31 (4.26, 4.37) | 0.66 (0.63, 0.69) |
| Comoros | 13.13 (11.85, 14.41) | 8.37 (7.51, 9.24) | 3.32 (2.51, 4.13) | 0.79 (0.18, 1.41) |
| Congo | 13.75 (13.12, 14.37) | 8.25 (7.83, 8.66) | 4.22 (3.82, 4.63) | 0.76 (0.44, 1.09) |
| Cook Islands | 17.39 (11.84, 22.95) | 11.77 (7.62, 15.93) | 4.22 (0.61, 7.83) | 0.80 (0.00, 2.97) |
| Costa Rica | 16.76 (16.45, 17.08) | 11.18 (10.94, 11.42) | 4.30 (4.10, 4.49) | 0.71 (0.60, 0.81) |
| Croatia | 15.88 (15.60, 16.17) | 8.45 (8.27, 8.64) | 5.27 (5.10, 5.44) | 1.67 (1.49, 1.85) |
| Cuba | 16.28 (16.13, 16.44) | 10.87 (10.75, 10.99) | 4.14 (4.04, 4.23) | 0.72 (0.67, 0.77) |
| Cyprus | 16.47 (15.58, 17.36) | 10.99 (10.38, 11.60) | 3.89 (3.22, 4.56) | 0.98 (0.86, 1.10) |
| Czechia | 16.36 (16.19, 16.52) | 8.53 (8.42, 8.65) | 5.61 (5.51, 5.71) | 1.76 (1.66, 1.86) |
| CÃ´te d'Ivoire | 13.62 (13.32, 13.92) | 8.91 (8.71, 9.12) | 3.29 (3.10, 3.48) | 0.77 (0.62, 0.91) |
| Democratic People's Republic of Korea | 15.51 (15.36, 15.67) | 11.71 (11.59, 11.83) | 2.73 (2.63, 2.83) | 0.42 (0.37, 0.48) |
| Democratic Republic of the Congo | 12.61 (12.46, 12.75) | 7.98 (7.88, 8.08) | 3.31 (3.22, 3.40) | 0.69 (0.62, 0.76) |
| Denmark | 16.36 (16.16, 16.57) | 10.71 (10.55, 10.87) | 3.93 (3.81, 4.05) | 1.10 (1.05, 1.15) |
| Djibouti | 13.56 (12.05, 15.07) | 8.35 (7.36, 9.35) | 3.82 (2.84, 4.81) | 0.78 (0.02, 1.54) |
| Dominica | 16.07 (13.66, 18.49) | 10.76 (8.89, 12.62) | 4.08 (2.58, 5.58) | 0.69 (0.00, 1.55) |
| Dominican Republic | 16.65 (16.39, 16.92) | 10.93 (10.74, 11.12) | 4.44 (4.26, 4.61) | 0.74 (0.63, 0.84) |
| Ecuador | 17.46 (17.25, 17.67) | 11.69 (11.53, 11.84) | 4.51 (4.37, 4.64) | 0.77 (0.69, 0.86) |
| Egypt | 13.33 (13.18, 13.48) | 8.99 (8.89, 9.09) | 3.13 (3.04, 3.22) | 0.52 (0.45, 0.59) |
| El Salvador | 16.28 (16.02, 16.54) | 10.93 (10.72, 11.13) | 4.11 (3.96, 4.27) | 0.66 (0.58, 0.74) |
| Equatorial Guinea | 14.25 (13.00, 15.50) | 8.64 (7.80, 9.49) | 4.21 (3.41, 5.01) | 0.86 (0.23, 1.49) |
| Eritrea | 11.87 (11.19, 12.55) | 7.72 (7.28, 8.15) | 2.85 (2.41, 3.30) | 0.64 (0.30, 0.97) |
| Estonia | 17.15 (16.69, 17.61) | 8.92 (8.61, 9.24) | 6.13 (5.85, 6.42) | 1.72 (1.46, 1.99) |
| Eswatini | 12.56 (11.63, 13.48) | 7.96 (7.32, 8.61) | 3.38 (2.80, 3.96) | 0.68 (0.22, 1.13) |
| Ethiopia | 14.49 (14.34, 14.65) | 8.20 (8.09, 8.30) | 4.99 (4.88, 5.10) | 0.81 (0.73, 0.88) |
| Fiji | 15.48 (14.32, 16.65) | 10.61 (9.77, 11.45) | 3.68 (2.89, 4.46) | 0.61 (0.14, 1.08) |
| Finland | 16.97 (16.77, 17.17) | 11.33 (11.17, 11.49) | 3.96 (3.84, 4.08) | 1.07 (1.02, 1.12) |
| France | 16.85 (16.80, 16.91) | 11.16 (11.11, 11.21) | 4.00 (3.97, 4.04) | 1.08 (1.07, 1.10) |
| Gabon | 14.44 (13.63, 15.25) | 8.89 (8.33, 9.46) | 4.09 (3.58, 4.60) | 0.90 (0.49, 1.30) |
| Gambia | 13.68 (12.84, 14.51) | 8.75 (8.17, 9.33) | 3.61 (3.07, 4.14) | 0.72 (0.34, 1.10) |
| Georgia | 15.19 (14.94, 15.44) | 7.24 (7.06, 7.42) | 6.00 (5.84, 6.16) | 1.46 (1.34, 1.59) |
| Germany | 16.92 (16.87, 16.97) | 11.28 (11.24, 11.32) | 3.98 (3.95, 4.02) | 1.06 (1.05, 1.07) |
| Ghana | 14.30 (14.07, 14.53) | 9.08 (8.93, 9.24) | 3.84 (3.69, 3.98) | 0.79 (0.68, 0.90) |
| Greece | 16.49 (16.34, 16.64) | 11.21 (11.09, 11.33) | 3.68 (3.58, 3.78) | 0.96 (0.92, 0.99) |
| Greenland | 13.93 (10.21, 17.65) | 8.07 (5.47, 10.67) | 4.32 (1.64, 6.99) | 0.84 (0.28, 1.40) |
| Grenada | 15.89 (13.84, 17.94) | 10.53 (8.94, 12.12) | 4.18 (2.92, 5.45) | 0.65 (0.00, 1.36) |
| Guam | 17.38 (15.79, 18.98) | 11.63 (10.35, 12.90) | 4.36 (3.40, 5.32) | 0.81 (0.28, 1.34) |
| Guatemala | 15.11 (14.79, 15.42) | 10.33 (10.12, 10.55) | 3.60 (3.38, 3.81) | 0.56 (0.44, 0.69) |
| Guinea | 12.44 (12.12, 12.75) | 8.47 (8.24, 8.70) | 2.61 (2.42, 2.80) | 0.67 (0.53, 0.80) |
| Guinea-Bissau | 12.15 (11.04, 13.25) | 8.19 (7.44, 8.94) | 2.71 (1.99, 3.42) | 0.61 (0.09, 1.12) |
| Guyana | 15.40 (14.43, 16.37) | 10.25 (9.53, 10.97) | 4.01 (3.39, 4.64) | 0.62 (0.25, 0.99) |
| Haiti | 13.28 (12.94, 13.61) | 9.23 (8.99, 9.46) | 2.98 (2.77, 3.20) | 0.47 (0.35, 0.60) |
| Honduras | 14.87 (14.50, 15.23) | 10.22 (9.96, 10.47) | 3.51 (3.27, 3.76) | 0.54 (0.40, 0.68) |
| Hungary | 16.17 (16.00, 16.34) | 8.47 (8.36, 8.59) | 5.53 (5.42, 5.63) | 1.72 (1.62, 1.81) |
| Iceland | 17.31 (16.40, 18.23) | 11.44 (10.70, 12.19) | 4.11 (3.57, 4.66) | 1.18 (0.96, 1.41) |
| India | 15.70 (15.67, 15.73) | 9.72 (9.70, 9.74) | 4.91 (4.89, 4.93) | 0.55 (0.54, 0.56) |
| Indonesia | 13.62 (13.55, 13.70) | 8.48 (8.43, 8.53) | 3.95 (3.89, 4.00) | 0.53 (0.50, 0.56) |
| Iran (Islamic Republic of) | 15.13 (15.01, 15.24) | 9.10 (9.03, 9.18) | 4.67 (4.60, 4.75) | 0.74 (0.68, 0.79) |
| Iraq | 14.25 (14.06, 14.43) | 9.25 (9.12, 9.38) | 3.60 (3.49, 3.71) | 0.69 (0.61, 0.78) |
| Ireland | 16.95 (16.69, 17.21) | 11.33 (11.12, 11.54) | 3.95 (3.79, 4.11) | 1.06 (1.00, 1.12) |
| Israel | 16.91 (16.71, 17.12) | 11.30 (11.14, 11.46) | 4.02 (3.90, 4.15) | 0.98 (0.94, 1.02) |
| Italy | 17.61 (17.55, 17.67) | 11.32 (11.27, 11.36) | 4.78 (4.74, 4.82) | 0.96 (0.94, 0.97) |
| Jamaica | 16.31 (15.94, 16.68) | 10.84 (10.54, 11.14) | 4.19 (3.97, 4.41) | 0.72 (0.61, 0.84) |
| Japan | 21.09 (21.05, 21.13) | 14.24 (14.20, 14.27) | 6.05 (6.02, 6.08) | 0.58 (0.57, 0.58) |
| Jordan | 15.36 (14.84, 15.88) | 9.75 (9.41, 10.09) | 4.16 (3.82, 4.49) | 0.79 (0.51, 1.06) |
| Kazakhstan | 16.43 (16.21, 16.64) | 7.31 (7.17, 7.44) | 7.33 (7.20, 7.45) | 1.44 (1.29, 1.59) |
| Kenya | 14.55 (14.36, 14.74) | 8.33 (8.20, 8.46) | 4.90 (4.77, 5.03) | 0.83 (0.74, 0.92) |
| Kiribati | 15.00 (10.87, 19.12) | 10.27 (7.33, 13.21) | 3.58 (0.80, 6.37) | 0.56 (0.00, 2.21) |
| Kuwait | 16.50 (15.84, 17.16) | 10.37 (9.89, 10.85) | 4.47 (4.07, 4.88) | 0.97 (0.65, 1.29) |
| Kyrgyzstan | 15.65 (15.22, 16.08) | 7.07 (6.79, 7.34) | 6.71 (6.46, 6.96) | 1.44 (1.16, 1.72) |
| Lao People's Democratic Republic | 12.40 (11.96, 12.84) | 8.37 (8.05, 8.69) | 2.78 (2.50, 3.06) | 0.48 (0.33, 0.63) |
| Latvia | 16.63 (16.27, 16.99) | 8.76 (8.51, 9.01) | 5.85 (5.63, 6.08) | 1.62 (1.42, 1.83) |
| Lebanon | 15.02 (14.74, 15.31) | 9.36 (9.15, 9.57) | 4.16 (3.99, 4.34) | 0.79 (0.67, 0.91) |
| Lesotho | 11.62 (11.11, 12.13) | 7.31 (6.94, 7.69) | 3.24 (2.93, 3.55) | 0.56 (0.35, 0.77) |
| Liberia | 13.59 (12.96, 14.23) | 8.95 (8.51, 9.39) | 3.24 (2.84, 3.65) | 0.77 (0.47, 1.06) |
| Libya | 14.84 (14.47, 15.22) | 9.44 (9.16, 9.71) | 3.99 (3.76, 4.22) | 0.74 (0.57, 0.91) |
| Lithuania | 16.57 (16.26, 16.88) | 8.73 (8.52, 8.94) | 5.83 (5.64, 6.02) | 1.61 (1.42, 1.79) |
| Luxembourg | 16.97 (16.30, 17.65) | 11.28 (10.74, 11.83) | 4.03 (3.62, 4.44) | 1.05 (0.89, 1.20) |
| Madagascar | 11.56 (11.26, 11.86) | 7.78 (7.57, 7.98) | 2.42 (2.23, 2.61) | 0.66 (0.52, 0.80) |
| Malawi | 11.99 (11.73, 12.25) | 7.94 (7.75, 8.13) | 2.72 (2.56, 2.88) | 0.68 (0.57, 0.80) |
| Malaysia | 14.22 (14.02, 14.43) | 9.10 (8.95, 9.24) | 3.85 (3.71, 3.98) | 0.58 (0.51, 0.66) |
| Maldives | 14.68 (12.74, 16.63) | 9.19 (7.79, 10.60) | 4.19 (2.92, 5.47) | 0.60 (0.00, 1.31) |
| Mali | 12.48 (12.08, 12.88) | 8.35 (8.09, 8.61) | 2.82 (2.56, 3.09) | 0.65 (0.45, 0.84) |
| Malta | 16.94 (16.21, 17.67) | 11.25 (10.66, 11.84) | 4.03 (3.58, 4.48) | 1.05 (0.88, 1.22) |
| Marshall Islands | 14.91 (6.92, 22.91) | 9.97 (4.31, 15.63) | 3.88 (0.00, 9.31) | 0.53 (0.00, 3.74) |
| Mauritania | 14.23 (13.62, 14.84) | 9.32 (8.90, 9.75) | 3.41 (3.02, 3.80) | 0.84 (0.55, 1.13) |
| Mauritius | 14.61 (14.05, 15.16) | 9.20 (8.77, 9.63) | 4.12 (3.77, 4.46) | 0.61 (0.42, 0.79) |
| Mexico | 17.25 (17.17, 17.33) | 11.00 (10.95, 11.06) | 5.08 (5.03, 5.14) | 0.71 (0.68, 0.74) |
| Micronesia (Federated States of) | 15.44 (11.71, 19.18) | 10.53 (7.80, 13.26) | 3.73 (1.27, 6.20) | 0.60 (0.00, 2.07) |
| Monaco | 17.05 (14.85, 19.24) | 11.41 (9.59, 13.22) | 3.94 (2.65, 5.23) | 1.11 (0.55, 1.67) |
| Mongolia | 16.05 (15.11, 17.00) | 6.99 (6.44, 7.55) | 7.31 (6.79, 7.84) | 1.38 (0.67, 2.08) |
| Montenegro | 15.19 (14.38, 16.01) | 8.18 (7.64, 8.71) | 5.18 (4.69, 5.67) | 1.35 (0.84, 1.86) |
| Morocco | 14.03 (13.87, 14.20) | 9.11 (9.00, 9.22) | 3.54 (3.44, 3.65) | 0.67 (0.59, 0.75) |
| Mozambique | 11.76 (11.54, 11.98) | 7.81 (7.65, 7.96) | 2.64 (2.50, 2.77) | 0.66 (0.56, 0.76) |
| Myanmar | 12.82 (12.71, 12.94) | 8.40 (8.32, 8.49) | 3.21 (3.13, 3.28) | 0.48 (0.45, 0.52) |
| Namibia | 12.81 (12.23, 13.39) | 8.03 (7.61, 8.45) | 3.51 (3.14, 3.87) | 0.68 (0.43, 0.94) |
| Nauru | 15.19 (0.10, 30.28) | 10.35 (0.00, 21.07) | 3.69 (0.00, 13.94) | 0.58 (0.00, 6.79) |
| Nepal | 13.21 (13.03, 13.38) | 8.99 (8.86, 9.12) | 3.00 (2.88, 3.11) | 0.51 (0.46, 0.57) |
| Netherlands | 16.99 (16.87, 17.11) | 11.40 (11.30, 11.49) | 3.95 (3.88, 4.03) | 1.05 (1.02, 1.08) |
| New Zealand | 18.54 (18.28, 18.81) | 11.88 (11.68, 12.09) | 5.24 (5.06, 5.43) | 0.94 (0.89, 0.99) |
| Nicaragua | 15.79 (15.37, 16.20) | 10.76 (10.46, 11.05) | 3.78 (3.50, 4.06) | 0.63 (0.47, 0.79) |
| Niger | 12.24 (11.86, 12.62) | 8.39 (8.14, 8.65) | 2.47 (2.22, 2.72) | 0.66 (0.48, 0.84) |
| Nigeria | 14.57 (14.47, 14.67) | 9.06 (8.99, 9.12) | 4.14 (4.08, 4.21) | 0.78 (0.74, 0.82) |
| Niue | 16.51 (0.00, 33.05) | 11.20 (0.00, 23.82) | 4.01 (0.00, 14.56) | 0.71 (0.00, 6.84) |
| North Macedonia | 14.40 (13.59, 15.20) | 7.93 (7.48, 8.39) | 4.72 (4.24, 5.20) | 1.22 (0.63, 1.82) |
| Northern Mariana Islands | 16.51 (11.29, 21.73) | 11.20 (7.43, 14.96) | 4.03 (0.53, 7.53) | 0.70 (0.00, 2.82) |
| Norway | 17.80 (17.58, 18.03) | 11.27 (11.09, 11.46) | 4.99 (4.84, 5.14) | 1.02 (0.97, 1.07) |
| Oman | 15.29 (14.47, 16.11) | 9.64 (9.08, 10.20) | 4.21 (3.69, 4.72) | 0.78 (0.37, 1.19) |
| Pakistan | 14.11 (14.02, 14.20) | 7.93 (7.86, 7.99) | 5.03 (4.97, 5.10) | 0.57 (0.54, 0.60) |
| Palau | 16.09 (8.31, 23.87) | 11.02 (5.38, 16.65) | 3.84 (0.00, 9.06) | 0.64 (0.00, 3.85) |
| Palestine | 14.46 (13.87, 15.05) | 9.18 (8.77, 9.60) | 3.92 (3.55, 4.29) | 0.68 (0.40, 0.95) |
| Panama | 16.56 (16.21, 16.92) | 10.99 (10.71, 11.26) | 4.31 (4.09, 4.54) | 0.67 (0.55, 0.79) |
| Papua New Guinea | 13.39 (12.92, 13.86) | 9.63 (9.28, 9.98) | 2.61 (2.31, 2.91) | 0.48 (0.31, 0.65) |
| Paraguay | 16.16 (15.87, 16.46) | 10.75 (10.52, 10.98) | 4.15 (3.97, 4.34) | 0.71 (0.60, 0.81) |
| Peru | 17.01 (16.89, 17.13) | 11.23 (11.14, 11.33) | 4.62 (4.54, 4.69) | 0.69 (0.65, 0.73) |
| Philippines | 13.29 (13.18, 13.41) | 8.01 (7.93, 8.09) | 4.04 (3.96, 4.12) | 0.54 (0.50, 0.58) |
| Poland | 17.09 (17.00, 17.19) | 8.58 (8.52, 8.65) | 6.28 (6.22, 6.34) | 1.86 (1.80, 1.91) |
| Portugal | 16.80 (16.66, 16.94) | 11.35 (11.23, 11.46) | 3.84 (3.75, 3.92) | 0.99 (0.96, 1.02) |
| Puerto Rico | 18.08 (17.82, 18.34) | 11.84 (11.63, 12.06) | 4.80 (4.63, 4.96) | 0.91 (0.82, 1.00) |
| Qatar | 16.12 (14.30, 17.94) | 10.24 (9.02, 11.46) | 4.32 (3.16, 5.47) | 0.89 (0.00, 1.87) |
| Republic of Korea | 21.20 (21.12, 21.28) | 15.26 (15.20, 15.33) | 5.03 (4.98, 5.09) | 0.66 (0.64, 0.67) |
| Republic of Moldova | 15.62 (15.34, 15.91) | 8.59 (8.38, 8.79) | 4.99 (4.82, 5.16) | 1.57 (1.41, 1.74) |
| Romania | 15.11 (14.98, 15.23) | 8.30 (8.22, 8.39) | 4.65 (4.58, 4.73) | 1.60 (1.53, 1.68) |
| Russian Federation | 16.85 (16.80, 16.90) | 8.42 (8.39, 8.45) | 6.58 (6.55, 6.61) | 1.54 (1.51, 1.57) |
| Rwanda | 12.23 (11.87, 12.60) | 8.22 (7.98, 8.46) | 2.56 (2.33, 2.78) | 0.75 (0.57, 0.93) |
| Saint Kitts and Nevis | 16.75 (13.69, 19.81) | 11.03 (8.73, 13.34) | 4.46 (2.51, 6.41) | 0.73 (0.00, 1.91) |
| Saint Lucia | 16.41 (14.89, 17.93) | 10.89 (9.73, 12.04) | 4.26 (3.30, 5.22) | 0.72 (0.15, 1.29) |
| Saint Vincent and the Grenadines | 16.17 (13.89, 18.45) | 10.76 (9.10, 12.43) | 4.16 (2.68, 5.65) | 0.70 (0.00, 1.60) |
| Samoa | 15.85 (13.51, 18.19) | 10.84 (9.10, 12.58) | 3.76 (2.24, 5.28) | 0.65 (0.00, 1.54) |
| San Marino | 17.23 (14.63, 19.83) | 11.48 (9.35, 13.61) | 4.05 (2.49, 5.62) | 1.08 (0.46, 1.71) |
| Sao Tome and Principe | 14.93 (12.11, 17.74) | 9.23 (7.31, 11.16) | 4.31 (2.48, 6.14) | 0.82 (0.00, 2.19) |
| Saudi Arabia | 15.46 (15.14, 15.79) | 9.81 (9.58, 10.03) | 4.19 (3.99, 4.40) | 0.81 (0.64, 0.98) |
| Senegal | 13.51 (13.22, 13.81) | 8.85 (8.65, 9.06) | 3.27 (3.09, 3.46) | 0.74 (0.60, 0.87) |
| Serbia | 15.17 (14.99, 15.34) | 8.27 (8.15, 8.39) | 4.84 (4.73, 4.94) | 1.53 (1.44, 1.63) |
| Seychelles | 14.65 (12.29, 17.00) | 9.25 (7.47, 11.03) | 4.11 (2.62, 5.60) | 0.61 (0.00, 1.42) |
| Sierra Leone | 13.02 (12.55, 13.50) | 8.57 (8.25, 8.90) | 3.10 (2.80, 3.41) | 0.70 (0.48, 0.91) |
| Singapore | 21.05 (20.78, 21.32) | 14.83 (14.61, 15.04) | 5.31 (5.13, 5.50) | 0.65 (0.60, 0.69) |
| Slovakia | 16.05 (15.81, 16.30) | 8.41 (8.25, 8.57) | 5.53 (5.39, 5.68) | 1.64 (1.50, 1.79) |
| Slovenia | 16.59 (16.23, 16.95) | 8.66 (8.41, 8.91) | 5.60 (5.38, 5.82) | 1.86 (1.65, 2.06) |
| Solomon Islands | 14.42 (12.17, 16.67) | 10.00 (8.44, 11.57) | 3.27 (1.74, 4.80) | 0.54 (0.00, 1.45) |
| Somalia | 10.80 (10.41, 11.19) | 7.31 (7.05, 7.57) | 2.30 (2.04, 2.55) | 0.55 (0.36, 0.74) |
| South Africa | 14.95 (14.84, 15.07) | 8.39 (8.31, 8.46) | 5.30 (5.23, 5.38) | 0.82 (0.77, 0.88) |
| South Sudan | 11.79 (11.34, 12.24) | 7.98 (7.67, 8.30) | 2.42 (2.15, 2.70) | 0.71 (0.50, 0.91) |
| Spain | 17.33 (17.26, 17.40) | 11.67 (11.61, 11.73) | 3.96 (3.92, 4.00) | 1.10 (1.08, 1.11) |
| Sri Lanka | 13.78 (13.60, 13.96) | 8.87 (8.74, 9.00) | 3.61 (3.49, 3.73) | 0.55 (0.49, 0.61) |
| Sudan | 13.37 (13.17, 13.58) | 8.84 (8.70, 8.99) | 3.18 (3.05, 3.30) | 0.63 (0.54, 0.72) |
| Suriname | 16.64 (15.71, 17.58) | 10.87 (10.16, 11.58) | 4.54 (3.94, 5.13) | 0.73 (0.38, 1.07) |
| Sweden | 15.88 (15.72, 16.04) | 9.85 (9.73, 9.97) | 4.42 (4.32, 4.52) | 0.91 (0.87, 0.94) |
| Switzerland | 16.76 (16.60, 16.92) | 11.14 (11.01, 11.28) | 4.01 (3.91, 4.11) | 0.98 (0.94, 1.02) |
| Syrian Arab Republic | 14.10 (13.56, 14.65) | 9.15 (8.80, 9.49) | 3.65 (3.29, 4.02) | 0.62 (0.32, 0.92) |
| Taiwan (Province of China) | 18.03 (17.90, 18.15) | 13.03 (12.93, 13.13) | 3.86 (3.78, 3.93) | 0.56 (0.53, 0.60) |
| Tajikistan | 14.10 (13.66, 14.54) | 6.62 (6.34, 6.90) | 5.75 (5.50, 6.01) | 1.22 (0.94, 1.50) |
| Thailand | 14.48 (14.40, 14.56) | 9.72 (9.67, 9.78) | 3.37 (3.32, 3.42) | 0.62 (0.60, 0.65) |
| Timor-Leste | 12.36 (11.19, 13.53) | 8.08 (7.25, 8.92) | 3.12 (2.34, 3.89) | 0.44 (0.03, 0.86) |
| Togo | 13.24 (12.73, 13.75) | 8.68 (8.34, 9.02) | 3.21 (2.88, 3.54) | 0.71 (0.46, 0.95) |
| Tokelau | 16.06 (0.00, 37.06) | 10.90 (0.00, 26.75) | 3.88 (0.00, 17.38) | 0.66 (0.00, 8.41) |
| Tonga | 16.05 (13.34, 18.77) | 11.11 (9.03, 13.20) | 3.60 (1.91, 5.30) | 0.70 (0.00, 1.68) |
| Trinidad and Tobago | 16.68 (16.16, 17.20) | 10.97 (10.57, 11.37) | 4.46 (4.13, 4.79) | 0.75 (0.56, 0.94) |
| Tunisia | 14.77 (14.55, 15.00) | 9.34 (9.18, 9.50) | 4.03 (3.89, 4.17) | 0.72 (0.62, 0.82) |
| Turkmenistan | 15.64 (15.29, 16.00) | 7.14 (6.89, 7.39) | 6.65 (6.44, 6.86) | 1.44 (1.24, 1.65) |
| Tuvalu | 15.50 (6.12, 24.88) | 10.52 (3.69, 17.35) | 3.79 (0.00, 10.02) | 0.60 (0.00, 4.23) |
| TÃ¼rkiye | 15.24 (15.16, 15.32) | 10.04 (9.98, 10.10) | 3.73 (3.68, 3.78) | 0.77 (0.74, 0.81) |
| Uganda | 12.35 (12.14, 12.55) | 8.14 (8.00, 8.29) | 2.78 (2.65, 2.91) | 0.74 (0.65, 0.84) |
| Ukraine | 16.32 (16.24, 16.40) | 8.31 (8.25, 8.36) | 6.16 (6.11, 6.22) | 1.48 (1.43, 1.53) |
| United Arab Emirates | 14.10 (12.37, 15.83) | 9.19 (8.05, 10.34) | 3.74 (2.61, 4.87) | 0.55 (0.00, 1.50) |
| United Kingdom | 18.13 (18.06, 18.19) | 11.86 (11.81, 11.91) | 4.66 (4.62, 4.70) | 1.10 (1.09, 1.12) |
| United Republic of Tanzania | 12.92 (12.76, 13.09) | 8.37 (8.26, 8.49) | 3.09 (2.99, 3.19) | 0.79 (0.71, 0.86) |
| United States Virgin Islands | 17.80 (15.61, 19.99) | 11.69 (10.12, 13.27) | 4.70 (3.23, 6.17) | 0.88 (0.00, 1.81) |
| United States of America | 18.65 (18.62, 18.68) | 11.54 (11.51, 11.56) | 5.55 (5.53, 5.57) | 1.20 (1.20, 1.21) |
| Uruguay | 17.06 (16.78, 17.33) | 11.35 (11.12, 11.57) | 4.29 (4.12, 4.47) | 0.86 (0.80, 0.92) |
| Uzbekistan | 15.85 (15.66, 16.04) | 7.21 (7.09, 7.33) | 6.73 (6.62, 6.84) | 1.49 (1.37, 1.62) |
| Vanuatu | 13.94 (11.07, 16.81) | 10.16 (8.12, 12.19) | 2.53 (0.62, 4.44) | 0.55 (0.00, 1.68) |
| Venezuela (Bolivarian Republic of) | 16.18 (16.06, 16.31) | 10.78 (10.69, 10.88) | 4.20 (4.12, 4.27) | 0.63 (0.59, 0.67) |
| Viet Nam | 12.84 (12.76, 12.92) | 8.50 (8.44, 8.56) | 3.07 (3.02, 3.12) | 0.50 (0.47, 0.52) |
| Yemen | 12.52 (12.27, 12.76) | 8.24 (8.07, 8.41) | 3.03 (2.88, 3.18) | 0.52 (0.42, 0.63) |
| Zambia | 12.22 (11.92, 12.51) | 7.94 (7.73, 8.15) | 2.99 (2.80, 3.18) | 0.67 (0.53, 0.81) |
| Zimbabwe | 11.10 (10.83, 11.36) | 7.44 (7.26, 7.63) | 2.47 (2.31, 2.63) | 0.57 (0.45, 0.69) |
